# Immunosuppressive biomaterial-based therapeutic vaccine to treat multiple sclerosis via re-establishing immune tolerance

**DOI:** 10.1038/s41467-022-35263-9

**Published:** 2022-12-02

**Authors:** Thanh Loc Nguyen, Youngjin Choi, Jihye Im, Hyunsu Shin, Ngoc Man Phan, Min Kyung Kim, Seung Woo Choi, Jaeyun Kim

**Affiliations:** 1 School of Chemical Engineering, Sungkyunkwan University (SKKU), Suwon, 16419 Republic of Korea; 2 Center for Theragnosis, Biomedical Research Institute, Korea Institute of Science and Technology (KIST), Seoul, 02792 Republic of Korea; 3 Department of Health Sciences and Technology, Samsung Advanced Institute for Health Sciences & Technology (SAIHST), Sungkyunkwan University (SKKU), Seoul, 06355 Republic of Korea; 4 Department of Ophthalmology, Seoul National University College of Medicine, Seoul National University Bundang Hospital, Seongnam, 13620 Republic of Korea; 5 Biomedical Institute for Convergence at SKKU (BICS), Sungkyunkwan University (SKKU), Suwon, 16419 Republic of Korea; 6 Institute of Quantum Biophysics (IQB), Sungkyunkwan University (SKKU), Suwon, 16419 Republic of Korea

**Keywords:** Multiple sclerosis, Biomaterials - vaccines, Autoimmune diseases, Regulatory T cells

## Abstract

Current therapies for autoimmune diseases, such as multiple sclerosis (MS), induce broad suppression of the immune system, potentially promoting opportunistic infections. Here, we report an immunosuppressive biomaterial-based therapeutic vaccine carrying self-antigen and tolerance-inducing inorganic nanoparticles to treat experimental autoimmune encephalomyelitis (EAE), a mouse model mimicking human MS. Immunization with self-antigen-loaded mesoporous nanoparticles generates Foxp3^+^ regulatory T-cells in spleen and systemic immune tolerance in EAE mice, reducing central nervous system-infiltrating antigen-presenting cells (APCs) and autoreactive CD4^+^ T-cells. Introducing reactive oxygen species (ROS)-scavenging cerium oxide nanoparticles (CeNP) to self-antigen-loaded nanovaccine additionally suppresses activation of APCs and enhances antigen-specific immune tolerance, inducing recovery in mice from complete paralysis at the late, chronic stage of EAE, which shows similarity to chronic human MS. This study clearly shows that the ROS-scavenging capability of catalytic inorganic nanoparticles could be utilized to enhance tolerogenic features in APCs, leading to antigen-specific immune tolerance, which could be exploited in treating MS.

## Abstract (web summary)

Multiple sclerosis is a debilitating autoimmune disease, for which therapy is not curative, only slowing down progression at the expense of general immune suppression. Here authors show that in a mouse model of multiple sclerosis, disease progression could be halted or even reversed by a nanovaccine, composed of reactive oxygen species scavenging cerium oxide nanoparticles, which establishes immune tolerance against the relevant autoantigen.

## Introduction

Multiple sclerosis (MS) is a neuroinflammatory disease of the brain and spinal cord where autoreactive CD4^+^ T cells invade the central nervous system (CNS) and recognize myelin as a foreign antigen, causing demyelination, axon damage, and motor disorder^1,2^. Current therapeutics can only temporarily ameliorate the severity of chronic pain caused by MS and potentially induce immunodeficiency^3^. Antigen-specific tolerance can downregulate effector T cells without causing global immunosuppression and is emerging as an alternative strategy to treat autoimmune diseases^4^. Many studies have focused on inducing forkhead box P3 (Foxp3)-expressing regulatory CD4^+^ T cells (Treg), which actively suppress the function of autoreactive CD4^+^ T cells against self-antigens^5–8^. The reduction in T-helper type 1 (Th1) and Th17, which accelerate MS development, is also known to facilitate disease suppression^9^.

The interaction between antigen**-**presenting cells (APC) and naive T cells in peripheral lymphoid organs, including the lymph nodes and spleen, predominantly determines the fate and immunological functions of T cells^10^. Two main signals are required to prime T cells: from cognate antigens on major histocompatibility complex (MHC) and costimulatory molecules (CD80, CD86, and CD40) on APCs^11^. It is generally accepted that tolerogenic APCs express medium or low levels of costimulatory molecules and secrete anti-inflammatory cytokines that convert naive T cells into Tregs and anergic T cells^12^. Recently, biocompatible nano/microparticles were utilized to deliver self-antigens with or without immunosuppressive agents to APCs, to polarize them to become tolerogenic for Treg generation^13–21^. However, most previous studies demonstrated the potential of biomaterials to induce immune tolerance to prevent or treat early-stage MS^14,18–20^.

Here, we describe an immunosuppressive therapeutic vaccine for the treatment of experimental autoimmune encephalomyelitis (EAE), a mouse model mimicking human MS, at late chronic phase. The high antigen**-**loading capacity of mesoporous silica nanoparticles (MSN) enables them to deliver a sufficient number of self-antigens to splenic APCs, even at a small dose. These MSNs induce tolerogenic APCs with moderate expression of costimulatory molecules. The induction of tolerogenic APCs and subsequent immune tolerance in EAE mice are enhanced by the presence of reactive oxygen species (ROS)-scavenging cerium oxide nanoparticles (CeNP) on the surface of self-antigen-loaded MSN vaccine. Consequently, the ROS-scavenging immunosuppressive nanoparticle vaccine generates a high number of Tregs in the peripheral lymphoid organ, suppresses the infiltration of autoreactive CD4^+^ T cells into the CNS, and suppresses EAE at a late chronic stage of disease in an antigen-specific manner.

## Results

### Myelin oligodendrocyte glycoprotein (MOG)-loaded MSNs suppresses EAE development in semi-therapeutic study

**Fig. 1:**
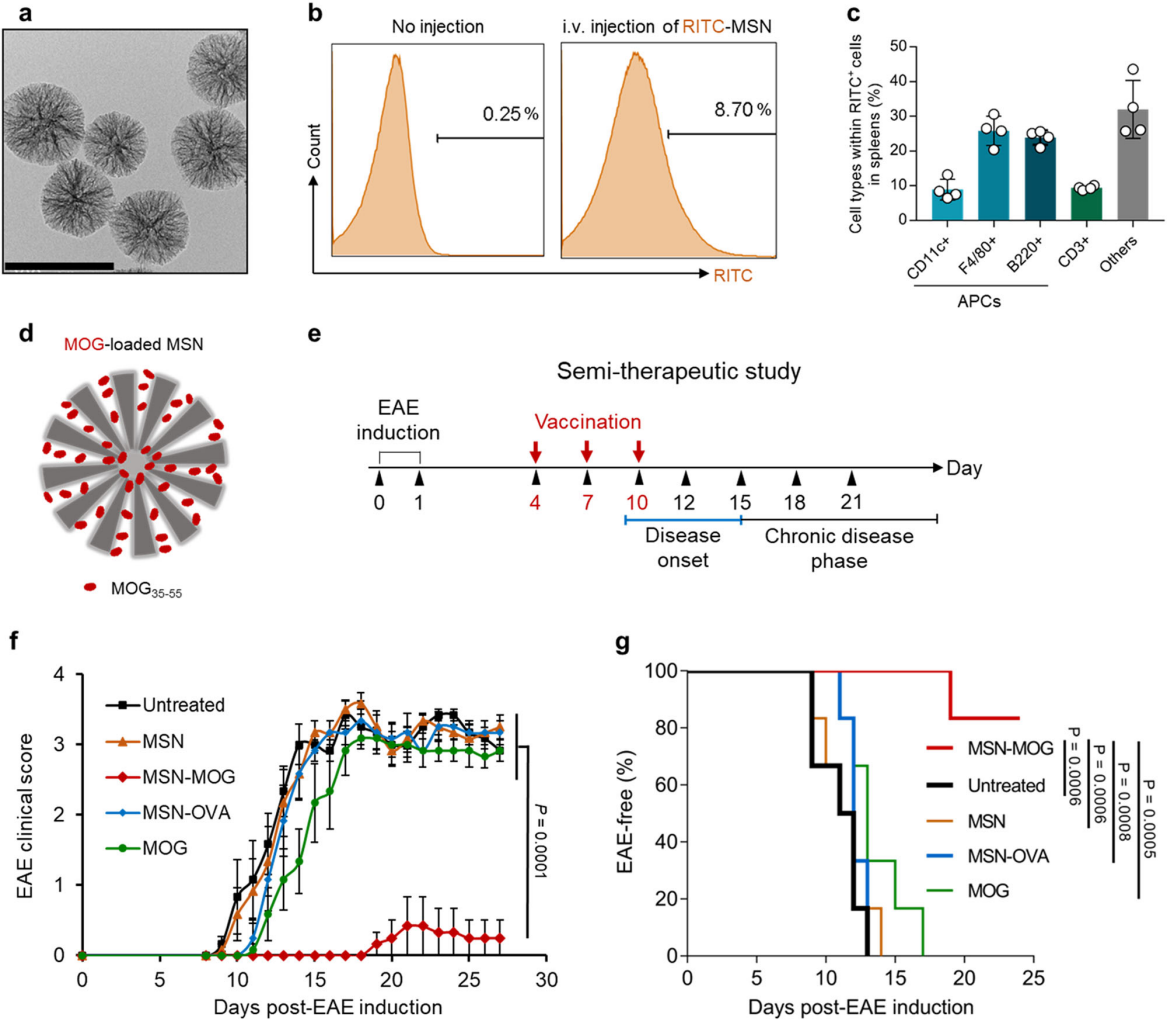
Intravenous injection of myelin oligodendrocyte glycoprotein (MOG)-loaded MSNs suppressed EAE development. **a** Transmission electron microscopy (TEM) image of MSNs, scale bar: 200 nm; the experiment was repeated independently at least three times. **b** Representative histograms of rhodamine B isothiocyanate (RITC) signal from splenocytes of C57BL/6 mice that were left untreated (no injection) or intravenously injected with RITC-MSNs 24 h before flow cytometry analysis. **c** Percentage of immune cells that engulfed RITC-MSNs in spleens, *n* = 4 biologically independent animals. **d** Schematics for MOG loading in MSNs. **e** EAE was induced in C57BL/6 mice before being injected intravenously with bare MSNs (MSN), MOG_35–55_ peptide**-**loaded MSNs (MSN**-**MOG), OVA_323–339_ peptide**-**loaded MSNs (MSN**-**OVA), soluble MOG (MOG), or left untreated on days 4, 7, and 10 (*n* = 6). **f** EAE clinical score for different groups of mice. **g** Kaplan–Meier curves demonstrating the percentage of EAE**-**free mice over time, *P* values were calculated using log**-**rank (Mantel–Cox) test. Data in (**c**) are represented as mean ± standard deviation (SD). Data in (**f**) are represented as mean ± standard error (SE). Data in (**f**) were subjected to one**-**way analysis of variance (ANOVA) with Dunnett’s multiple comparisons test. *P* < 0.05 was considered significant.

First, MSNs with 10~30**-**nm large mesopores along with 3-nm conventional mesopores were synthesized according to previous reports^22,23^ (Fig. 1a and Supplementary Fig. 1). The degradation of MSNs with amorphous silica wall in phosphate-buffered saline (PBS) at physiological pH (Supplementary Fig. 2a) was observed, which is derived from hydrolysis of siloxane bonds in its amorphous silica wall^24,25^. The cytotoxicity of various concentrations of MSNs was examined, showing that the MSNs were highly biocompatible (Supplementary Fig. 2b). In line with previous studies^22,26^, we first demonstrated that the systemic administration of MSNs led to the accumulation of nanoparticles in the spleen (Fig. 1b), which is attributed to the intrinsic capacity of the mononuclear phagocytic system to massively capture foreign nanoparticles^27,28^. APCs, including CD11c^+^ dendritic cells (DCs), F4/80^+^ macrophages, and B220^+^ B cells were the major cell populations that engulfed MSNs in the spleen (Fig. 1c). According to our previous report, the systemic administration of MSNs did not cause a “cytokine storm” or hepatotoxicity, verifying the in vivo applicability of MSNs^22^. Owing to their large pore size, MSNs used in this study exhibited high antigen-loading capacity; thus, large-pore MSNs could carry antigen (MOG_35–55_ peptide), equivalent to that used in previous studies^14,18,20^ at a substantially lower dose (Fig. 1d and Supplementary Fig. 3).

We directly tested our hypothesis in EAE-induced C57BL/6 mice (Fig. 1e). Intravenous injection of bare MSNs into EAE-induced mice prior to disease onset (days 4, 7, and 10; semi-therapeutics) was unable to prevent typical EAE progression (Fig. 1f). Injecting soluble MOG slightly delayed EAE progression, whereas MOG-loaded MSNs (MSN-MOG) markedly impeded EAE development, resulting in a high percentage of healthy animals (Fig. 1g). These results indicate that loading antigens on MSNs is critical for suppressing EAE development. Conversely, the OVA_323–339_ peptide (unrelated peptide antigen from ovalbumin)-loaded MSNs (MSN-OVA) did not suppress EAE development, indicating that the reduction in disease severity induced by MSN-MOG was antigen-specific. Meanwhile, the increase in nanoparticle amount when maintaining MOG peptide dose was unable to show comparable benefits (Supplementary Fig. 4).

### MOG-loaded MSN vaccine generates induced Tregs and antigen-specific tolerogenic immune responses

**Fig. 2:**
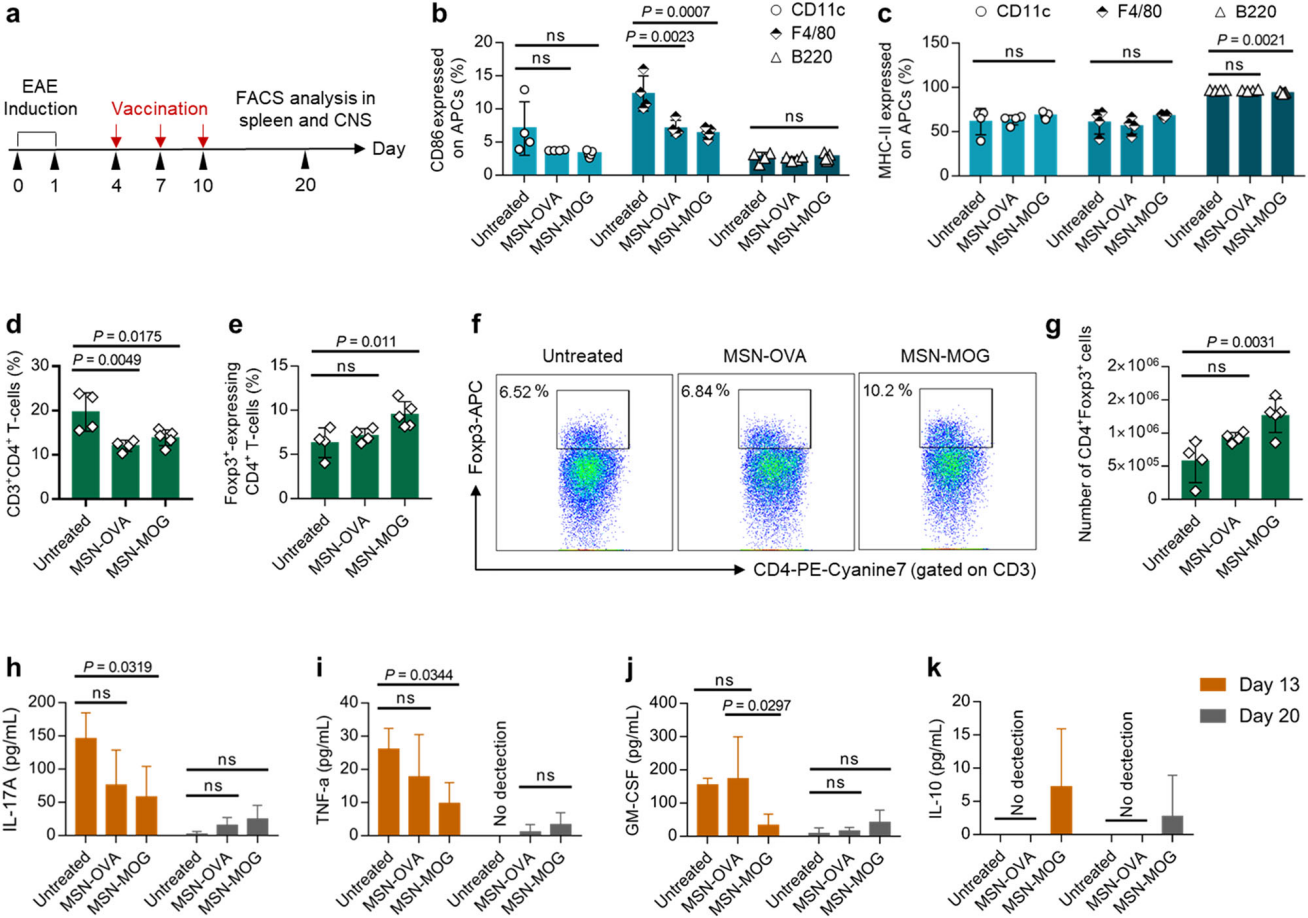
MSN-MOG induced peripheral tolerance in the spleen. **a** EAE**-**induced mice were intravenously injected with MSN**-**OVA, MSN**-**MOG, or left untreated on days 4, 7, and 10 after EAE induction. Splenocytes were isolated and analyzed by flow cytometry on day 20 after EAE induction. **b**, **c** CD86 and MHC**-**II expression on CD11c^+^DCs, F4/80^+^ macrophages, and B220^+^ B cells, respectively. **d** Frequency of CD4^+^ T cells. **e**, **f** Percentage of forkhead box P3^+^ (Foxp3^+^) among CD4^+^ T cells and their representative pseudocolor plots, respectively. **g** Number of Foxp3^+^ regulatory T cells (Tregs). **h**–**k** Levels of IL-17A, TNF**-**α, GM**-**CSF, and IL-10 secreted by splenocytes stimulated ex vivo with MOG_35–55_, respectively. In (**b**–**e**, **g**–**k**), *n* = 4 (untreated and MSN**-**OVA) or 5 (MSN**-**MOG) biologically independent animals. Data in (**b**–**e**, **g**–**k**) are represented as mean ± SD and were subjected to a one**-**way ANOVA with Dunnett’s multiple comparisons tests. *P* < 0.05 was considered significant, ns = not significant.

We evaluated cellular responses in EAE-induced mice after MSN-MOG injection to gain a better insight into the mechanism underlying immune tolerance. Phenotypic alterations in CD11c^+^ DCs, F4/80^+^ macrophages, and B220^+^ B cells in the spleen, were examined by flow cytometry after vaccination (Fig. 2a). CD86 expressions on F4/80^+^ macrophages were significantly lower in the MSN-MOG group than those in the untreated group (Fig. 2b). We observed no significant difference in the activation of MHC class II (MHC-II) on APCs between the three groups (Fig. 2c); indicating that antigen presentation by MHC-II was not affected by immunization. The number of APCs increased (Supplementary Fig. 5), while the expression of activation markers on APCs did not increase significantly. These data suggest that systemic administration of peptide antigen (MOG)-loaded MSNs did not promote APC maturation in the lymphoid organ in EAE mice, which is necessary for the polarization of naive T cells into Tregs and/or anergic T cells.

Given the inability of MSN-MOG to activate splenic APCs in EAE mice, we further examined whether MSN-MOG administration could induce Foxp3^+^ Tregs. Following treatment with peptide-loaded MSNs, we observed a decrease in the percentage of splenic CD4^+^ T cells on day 20 (Fig. 2d). In contrast, there were noticeable increases of both frequencies and numbers of Foxp3^+^ Tregs in mice vaccinated with MSN-MOG (Fig. 2e–g). These observations suggest that the systemic delivery of self-antigen (MOG)-loaded MSNs modified the cellular composition of the spleen into more tolerogenic T cell population in the EAE mice, which is a desirable change to treat EAE.

We next evaluated the responsiveness of immune cells retrieved from spleen upon re-stimulation with EAE-associated antigens (MOG) at different time points (days 13 and 20 after EAE induction, corresponding to days 3 and 10 after the last semi-therapeutic intervention) to confirm whether an antigen-specific tolerogenic environment was established. Interleukin 17A (IL-17A) and tumor necrosis factor-α (TNF-α), the central mediators of EAE and MS progression^29,30^, were significantly lower in the MSN-MOG-treated group than in the untreated group (Fig. 2h, i). Similarly, granulocyte-macrophage colony-stimulating factor (GM-CSF) production, a key recruitment factor that attracts APCs and pathogenic monocyte-derived cells to the CNS^31^, was significantly inhibited in the MSN-MOG-treated group (Fig. 2j). We detected IL-10 in the MSN-MOG-injected group, but not in the untreated or MSN-OVA groups (Fig. 2k). IL-10 is a potent anti-inflammatory cytokine that plays a vital role in hampering immune responses against self-antigens^32^.

**Fig. 3:**
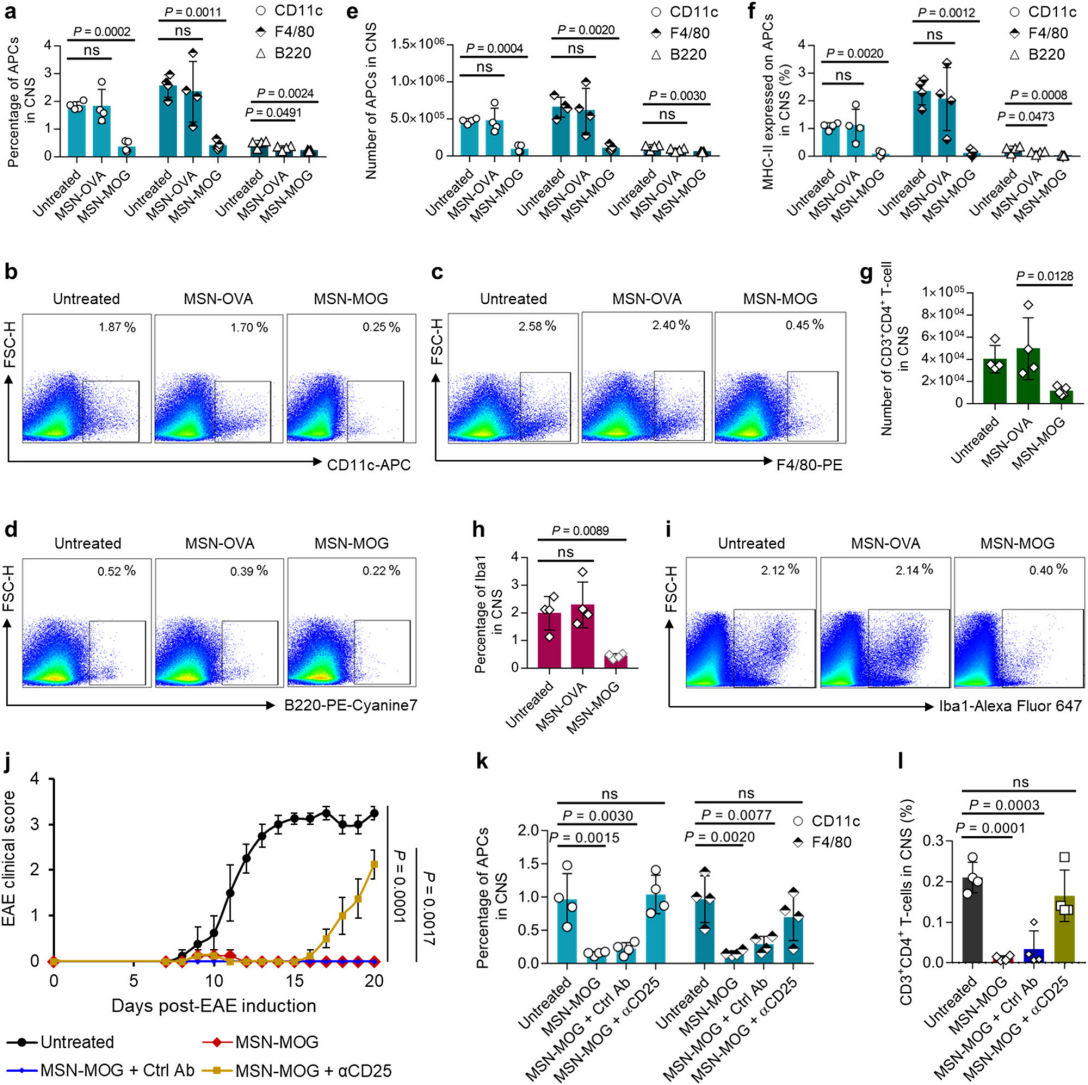
MSN-MOG vaccine suppressed CNS-infiltrating APCs and CD4^+^ T cells. EAE-induced mice were intravenously injected with MSN-OVA, MSN-MOG, or left untreated on days 4, 7, and 10 after EAE induction; *n* = 4 (untreated and MSN-OVA) or 5 (MSN-MOG) biologically independent animals. Cells from spinal cords were isolated and analyzed by flow cytometry on day 20 after EAE induction. **a** Percentage of CD11c^+^ DCs, F4/80^+^ macrophages, and B220^+^ B cells in the spinal cords and **b**–**d** their representative pseudocolor plots, respectively. **e** Number of CD11c^+^ DCs, F4/80^+^ macrophages, and B220^+^ B cells in spinal cords. **f** Percentage of APCs expressing MHC-II in spinal cords. **g** Number of CD4^+^ T-cells infiltrating spinal cords. **h**, **i** Percentage of Iba1 expressed in spinal cord cells and representative pseudocolor plots, respectively. **j** EAE mean clinical score of EAE mice that were injected with MSN-MOG, MSN-MOG and control antibody (MSN-MOG + Ctrl Ab), MSN-MOG and anti-CD25 antibody (MSN-MOG + αCD25), or left untreated (*n* = 4). **k**, **l** The percentage of APCs and CD4^+^ T cells in the spinal cord on day 20 after disease induction, respectively. Data in (**a**, **e**–**h**, **k**, **l**) are represented as mean ± SD. Data in (**j**) are represented as mean ± SE. Data in (**a**, **e**–**h**, **j**–**l**) were subjected to a one-way ANOVA with Dunnett’s multiple comparisons tests. *P* < 0.05 was considered significant, ns = not significant.

To assess immune tolerance at the disease site, we characterized the disease-associated immune cells in the CNS of EAE-induced mice intravenously injected with MSN-MOG, MSN-OVA, or left untreated. Frequencies (Fig. 3a–d) and numbers (Fig. 3e) of CNS-infiltrating APCs decreased in the MSN-MOG-treated group. It was previously shown that macrophage-depleted mice significantly inhibit EAE^33^. The percentage (Fig. 3f) and the number (Supplementary Fig. 6) of CD11c^+^MHC-II^+^, F4/80^+^MHC-II^+^, and B220^+^MHC-II^+^ cells in the CNS decreased in MSN-MOG-injected mice. CD11c^+^ DCs expressing MHC-II could reactivate primed T cells and initiate EAE^34^ as DCs within inflamed CNS encounter myelin epitopes and present them to the infiltrated T cells^35^. The resulting self-reactive T cells contribute to epitope spreading in the CNS^36^. A decrease in the frequency and number of APCs in the CNS after MSN-MOG treatment might reduce T-cell responses in the CNS as there would be less antigen processing by APCs. Since the induced Tregs in secondary lymphoid organs (Fig. 2e–g) suppress a generation of autoreactive T cells, the number of autoreactive T cells migrating into the CNS may be decreased. Consistently, CD4^+^ T-cell infiltration in the CNS was significantly inhibited in the MSN-MOG group (Fig. 3g). Moreover, the expression of ionized calcium-binding adapter molecule 1 (Iba1), a marker of macrophage and microglia, in the cells retrieved from CNS was also notably suppressed by MSN-MOG vaccination (Fig. 3h, i), presenting that the MSN-MOG vaccination could suppress the pathological cellular responses of multiple sclerosis^37^.

As we have observed the increase of Tregs after vaccination in EAE mice (Fig. 2e–g), we further investigated whether the depletion of Tregs via anti-Treg antibody administration over the vaccination process would inhibit the therapeutic efficacy of MSN-MOG vaccine. The results showed that the depletion of Tregs in MSN-MOG-vaccinated mice diminished the therapeutic effect of MSN-MOG vaccine, and thus could not prevent EAE development (Fig. 3j). In addition, the cellular analysis revealed that depletion of Tregs led to the infiltration of CD11c^+^ cells, F4/80^+^ cells, and CD4^+^ T cells into the CNS (Fig. 3k, l and Supplementary Fig. 7). Taken together, these data (Figs. 2 and 3) demonstrate the potency of self-antigen-loaded MSNs in inducing systemic antigen-specific tolerance via Treg generation and reduction of Th1 and Th17-biased inflammatory cytokine secretion.

### MSN-MOG vaccine reduces disease severity following therapeutic intervention at the late stage of the disease

**Fig. 4:**
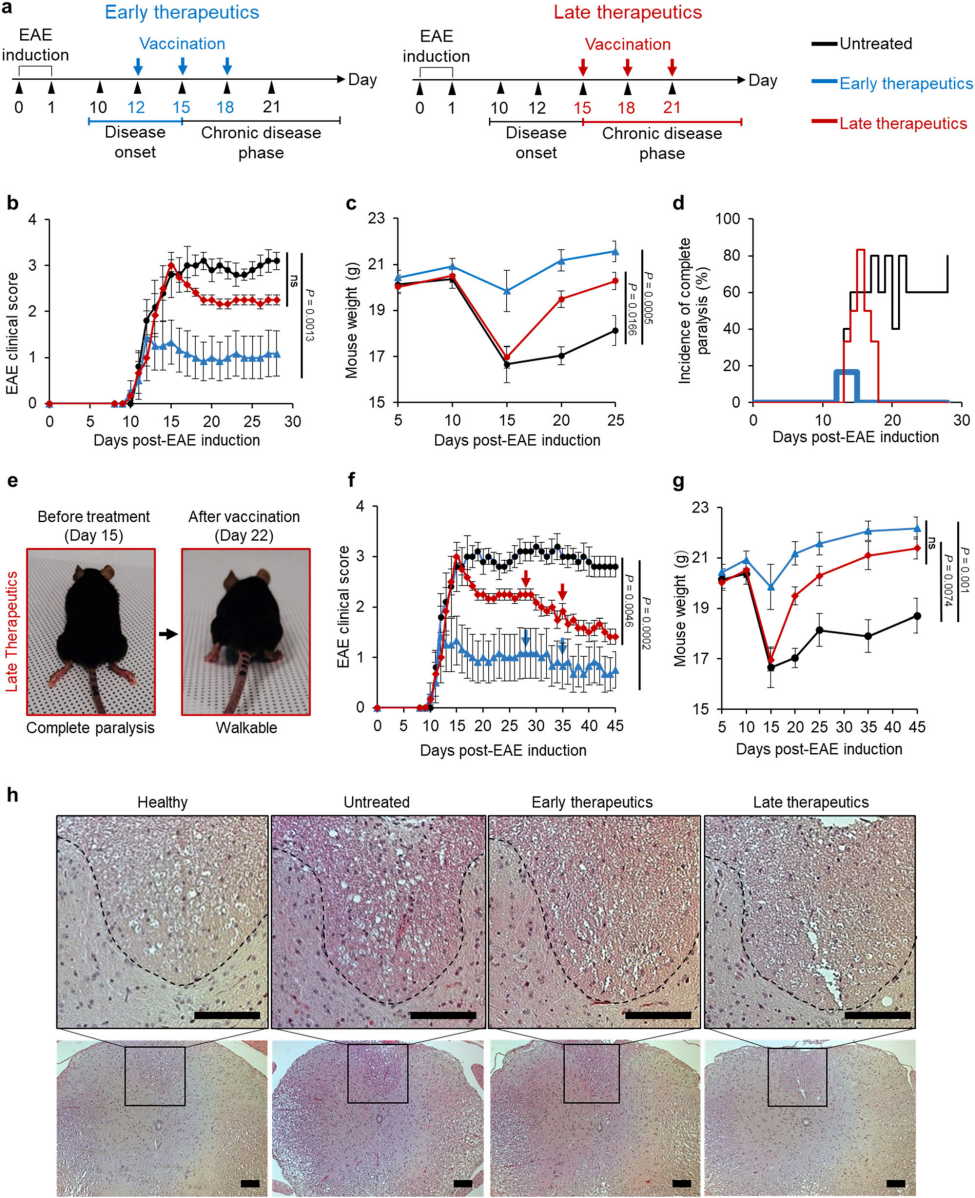
MSN-MOG vaccine therapeutically suppressed the developed EAE. **a** EAE was induced in mice before administering three MSN-MOG injections starting from day 12 (early therapeutics), day 15 (late therapeutics), or left untreated. **b**–**d** The clinical score, body weight of the mice, and incidence of complete paralysis, respectively, were measured over time. *n* = 5 (untreated) or 6 (early therapeutics, late therapeutics) biologically independent animals. **e** Photograph of an EAE-induced mouse from the late therapeutic group before receiving an injection on day 15 (left) and after 3 MSN-MOG injections on day 22. **f**, **g** The EAE clinical score and body weight of mice that received additional MSN-MOG injections on days 28 and 35, respectively; *n* = 5 (untreated) or 6 (early therapeutics, late therapeutics) biologically independent animals. **h** Images of Hematoxylin and eosin (H&E)-stained thoracic vertebrae cross sections of EAE-induced mice on day 50, the experiment was repeated independently at least twice. The discontinuous lines indicate the border between the gray matter (on the lower part) and the ventral white matter of the spinal cord (scale bar: 100 µm). The data in (**b**, **c**, **f**, and **g**) are represented as mean ± SE and were analyzed by one-way ANOVA. Dunnett’s multiple comparisons tests were performed in (**b**, **c**, and **f**). Tukey’s multiple comparisons test was performed in (**g**). *P* < 0.05 was considered significant, ns = not significant.

Although we have shown that MSN-MOG could suppress the development of EAE in semi-therapeutic study (Fig. 1f, g), MS in human is initiated before clinical symptoms appear, leading to the high demand for therapeutics to treat MS after the clinical diagnosis. Next, we investigated whether MSN-MOG suppressed EAE during the onset and chronic phases. EAE-induced mice received three intravenous MSN-MOG injections at 3 days intervals, starting on day 12 (the point of intermediate disease severity, early therapeutics) or day 15 (the peak of disease severity, late therapeutics) (Fig. 4a). The results showed that for both treatment regimens, progression of clinical episodes was strongly hampered immediately after the first injection (Fig. 4b), leading to body weight recovery of the mice (Fig. 4c). In both treated groups, complete paralysis disappeared three days after the first injection (Fig. 4d). For example, mice completely paralyzed on day 15 after EAE induction were able to walk on day 22 after vaccinations on days 15, 18, and 21 (Fig. 4e and Supplementary Movie 1). These data reveal that MSN-MOG mediated functional recovery from neuroinflammation in EAE.

As the clinical scores remained stable after three shots in both early and late therapeutic studies (Fig. 4b), we examined whether motor impairment could be further ameliorated by providing additional vaccinations on days 28 and 35, to all mice except the untreated group. EAE severity in the early and late therapeutic groups reduced strongly after two more shots (Fig. 4f). Especially, in late therapeutics group, the clinical score became significantly lower than in the untreated group, indicating the significance of therapeutic efficacy of nanovaccine to treat the late chronic stage of EAE. At the end of the study, the body weights of mice had recovered completely, and there were no differences between the early and late therapeutic groups (Fig. 4g). Representative hematoxylin and eosin (H&E)-stained histological images revealed fewer inflammatory cells in the ventral area of the white matter of MSN-MOG-treated mice in both early and late therapeutics groups than that of the untreated mice, indicating that the vaccination induced an improvement of neuro-immune homeostasis comparable to the healthy mice (naive mice) (Fig. 4h).

### ROS-scavenging CeNPs suppresses activation of APCs and induces tolerogenic APCs

**Fig. 5:**
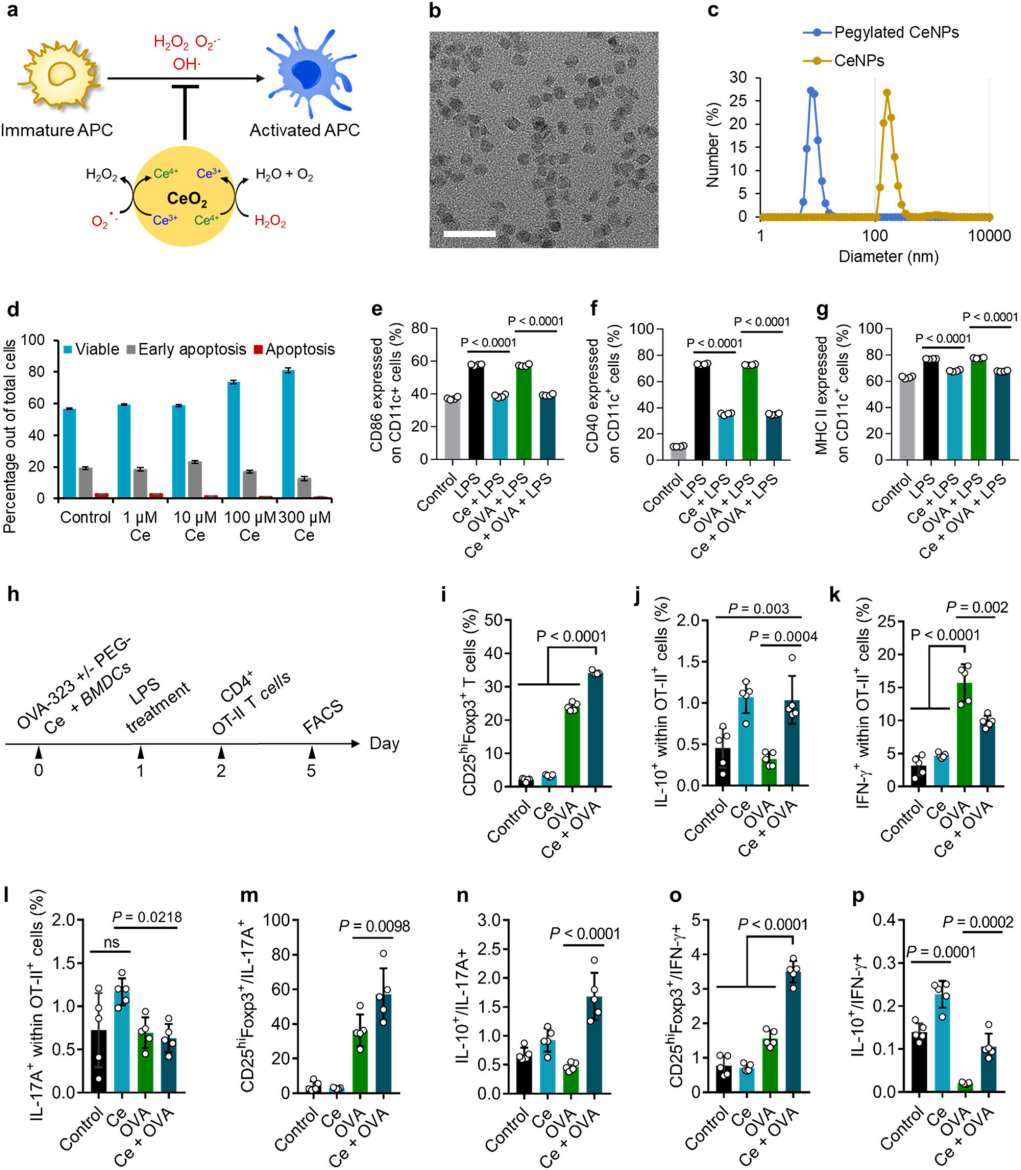
ROS-scavenging CeNPs induced tolerogenic APCs. **a** Scheme demonstrates the catalytic property of CeNPs to scavenge intracellular ROS for the suppression of APCs activation. **b** TEM images of CeNPs (scale bar: 20 nm), the experiment was repeated independently at least three times. **c** Hydrodynamic size distribution of CeNPs and pegylated CeNPs in complete RPMI 1640 medium. **d** Apoptosis of BMDCs after 48 h incubation with various cerium concentrations. BMDCs were incubated with pegylated CeNPs (Ce, 50 µM cerium), OVA_323–339_ (OVA, 1 µg/mL), pegylated CeNPs plus OVA_323–339_ (Ce + OVA), or left untreated for 24 h; following by LPS treatment (1 µg/mL) for the next 24 h; control is the bare BMDCs; *n* = 3 biologically independent samples. **e**–**g** Expression of CD86, CD40, and MHC-II on BMDCs, respectively; *n* = 4 biologically independent samples. **h** BMDCs were incubated with pegylated CeNPs (Ce, 50 µM cerium), OVA_323–339_ (OVA, 1 µg/mL), pegylated CeNPs plus OVA_323–339_ (Ce + OVA), or left untreated for 24 h; following by LPS treatment (1 µg/mL) for the next 24 h before being co**-**cultured with OT-II CD4^+^ T cells for 72 h. **i**–**l** The percentage of CD25^high^Foxp3, IL-10, IFN-γ, and IL-17A expression in the gate of Vα2^+^CD4^+^ T cells, respectively. **m**–**p** Ratios of CD25^high^Foxp3^+^ to IL-17A^+^ T cells, of IL-10^+^ to IL-17A^+^ T cells, of CD25^high^Foxp3^+^ to IFN-γ^+^ T cells, and of IL-10^+^ to IFN-γ^+^ T cells, respectively. In (**i**–**p**), *n* = 5 biologically independent samples. The data in (**d**–**g**, **i**–**p**) are represented as mean ± SD and were analyzed by one-way ANOVA with Tukey’s multiple comparisons test. *P* < 0.05 was considered significant, ns = not significant.

Since oxidative stress derived from high intracellular ROS is known to activate APC, especially in MS^38,39^, we hypothesized that scavenging intracellular ROS in APCs would further suppress their activation and potentially enhance the tolerogenic phenotype of APCs (Fig. 5a). The ceria nanoparticles (CeNPs) were recently engineered for the treatment of ROS-associated diseases, due to their ROS-scavenging catalytic properties that mimic catalase and superoxide dismutase;^40–45^ however, the immunological impact of these nanoparticles has not been investigated intensively. Water-dispersible, positively charged CeNPs (4 nm) were synthesized according to our previous reports (Fig. 5b)^46^. To improve the dispersion of CeNPs in the cell culture medium, polyethylene glycol (PEG) was coated on the CeNPs (Fig. 5c and Supplementary Fig. 8a), and the successful PEG modification was confirmed by FTIR spectra and zeta potential (Supplementary Fig. 8b, c). The resulting CeNPs were highly biocompatible (Fig. 5d) and were directly used to examine the capacity to suppress the activation of bone marrow-derived dendritic cells (BMDCs). BMDCs were cultured with CeNPs and treated with lipopolysaccharides (LPS), a TLR4 agonist known to induce excessive intracellular ROS production and enhance activation of APCs, resulting in a substantially decreased expression levels of costimulatory molecules (CD86 and CD40) and MHC-II (Fig. 5e–g). These data demonstrate the ROS-scavenging CeNPs could prevent activation of DCs and maintain the tolerogenic phenotype of DCs even under the presence of immune-activating TLR4 agonist.

We next evaluated whether the OVA_323–339_ peptide-experienced semi-mature DCs induced by CeNP could trigger the CD4^+^ T-cell differentiation to Tregs by co-culturing these DCs with CD4^+^ T cells retrieved from OT-II transgenic mice (Fig. 5h). The CD4^+^ T cells in OT-II mice have T-cell receptors that specifically recognize OVA_323–339_ peptide. A significantly higher percentage of CD25^+^Foxp3^+^ Tregs appeared in the CeNP + OVA-treated group than the others (Fig. 5i and Supplementary Fig. 9). Interestingly, the elevated population of IL-10 secreting CD4^+^ T cells, known as type 1 regulatory T cells (Tr1) that inhibit DC maturation and establish T-cell tolerance^47^, was observed in CeNP-treated group and the CeNP + OVA-treated group (Fig. 5j and Supplementary Fig. 9). In contrast, the IFN-γ secreting CD4^+^ T cells (activated Th1 cells) was significantly suppressed in CeNP + OVA-treated group compared to OVA-treated one (Fig. 5k and Supplementary Fig. 9). Although there was no difference in IL-17A secretion within CD4^+^ T cells between the OVA-treated group and the CeNP + OVA-treated group (Fig. 5l and Supplementary Fig. 9), the ratios of CD25^+^Foxp3^+^/IL-17A^+^ and IL-10^+^/IL-17A^+^ in CeNP + OVA-treated group were highest among groups (Fig. 5m, n). In addition, the bias of naive CD4^+^ T cells into Tregs and Tr1 rather than Th1 by CeNPs was demonstrated by the greater ratios of CD25^+^Foxp3^+^/IFN-γ^+^ and IL-10^+^/IFN-γ^+^ in the CeNP + OVA-treated group, respectively (Fig. 5o, p). Taken together, these data show that CeNPs could induce tolerogenic DCs which can skew naive CD4^+^ T cells toward Tregs and Tr1 in vitro, representing that CeNPs could be used as an immunosuppressive agent to enhance immune tolerance.

### ROS-scavenging MSN-MOG vaccine enhances the therapeutic efficacy in the late chronic phase of EAE

**Fig. 6:**
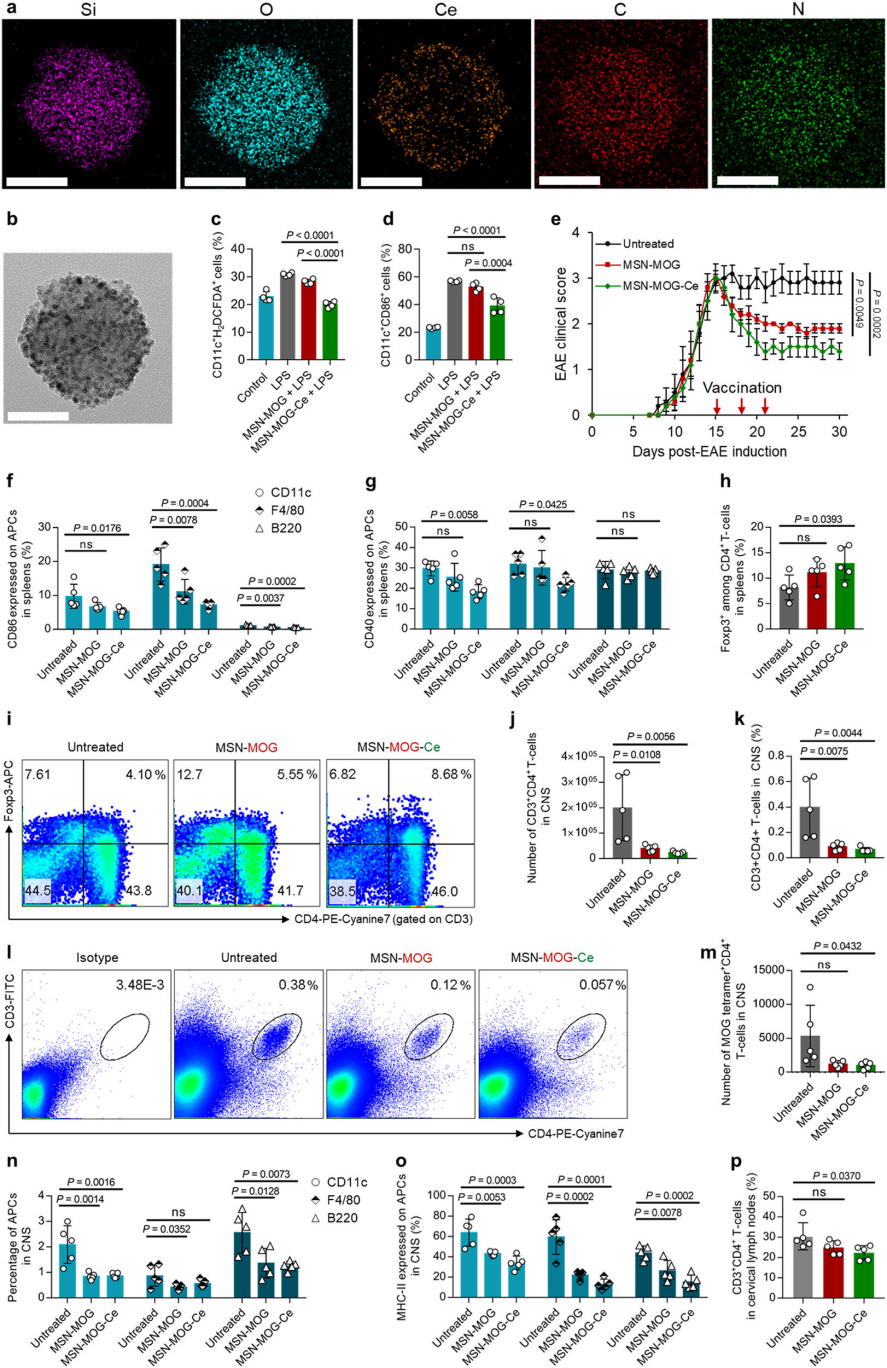
CeNPs-decorated MSN-MOG enhanced efficacy of the therapeutic vaccine against EAE at chronic phase. **a** Energy dispersive X-ray spectroscopy (EDS) elemental mapping and **b** the corresponding TEM image of MSN-MOG-Ce vaccine, scale bar: 50 nm; the experiment was repeated independently at least twice. BMDCs were treated with MSN-MOG, MSN-MOG-Ce, or left untreated for 24 h, followed by stimulation with LPS (100 ng/mL) for the next 12 h. **c** Intracellular ROS levels in BMDCs, as measured by H_2_DCFDA assay, *n* = 4 biologically independent samples. **d** The percentage of CD11c^+^ BMDCs expressing CD86, *n* = 4 biologically independent samples. **e** EAE clinical scores of mice that were intravenously injected with MSN-MOG, MSN-MOG-Ce, or left untreated on days 15, 18, and 21 after disease induction; *n* = 5 biologically independent animals. **f**, **g** The expression levels of CD86 and CD40 on APCs in spleens on day 31, respectively. **h**, **i** The percentage of Foxp3^+^ cells among CD3^+^ CD4^+^ T cells in spleens on day 31 and their representative contour plots, respectively. **j**, **k** The number and frequency of CD3^+^CD4^+^ T cells infiltrating the spinal cords on day 31. **l** Representative pseudocolor plots showing the frequency of CD3^+^CD4^+^ T cells in the spinal cord of the indicated groups. **m** The number of MOG-specific CD4^+^ T cells infiltrating to the spinal cord on day 31. **n**, **o** The percentage of APCs in the spinal cord and MHC-II expression on these cells on day 31, respectively. **p** The frequency of CD3^+^CD4^+^ T cells in cervical lymph nodes on day 31. In (**f**–**h**, **j**, **k**, **m**–**p**), *n* = 5 biologically independent animals. The data in (**c**, **d**, **f**–**h**, **j**, **k**, **m**–**p**) are represented as mean ± SD and were analyzed by one-way ANOVA. Tukey’s multiple comparisons tests were performed in (**c**, **d**). Dunnett’s multiple comparisons tests were performed in (**f**–**h**, **j**, **k**, **m**–**p**). The data in (**e**) are represented as mean ± SE and were subjected to a one-way ANOVA with Dunnett’s multiple comparisons test. *P* < 0.05 was considered significant, ns = not significant.

To enhance the therapeutic efficacy of autoimmune disease nanovaccine, CeNPs were additionally attached on MSN**-**MOG via electrostatic interactions between the negatively charged MSN**-**MOG and positively charged CeNPs (Supplementary Fig. 10a), resulting in the ROS**-**scavenging MSN**-**MOG**-**Ce nanovaccine (Fig. 6a, b). The attached CeNPs was stably maintained on the surface of MSN even after incubation in PBS up to 3 days (Supplementary Fig. 11). Furthermore, sustained release of MOG from the nanovaccine was observed while a minimal amount of CeNPs could be detached from MSN**-**MOG**-**Ce (Supplementary Fig. 12), which would be beneficial to deliver both self-antigen and ROS**-**scavenging nanoparticles into APCs in spleen without their significant loss after intravenous administration. We next examined the ability of MSN**-**MOG**-**Ce to scavenge intracellular ROS in BMDCs in the presence of LPS. The level of intracellular ROS was significantly lower in MSN**-**MOG**-**Ce**-**treated BMDCs than in LPS**-** and MSN**-**MOG**-**treated BMDCs (Fig. 6c), revealing that MSN**-**MOG**-**Ce attenuated oxidative stress in BMDCs under inflammatory conditions. Consistently, BMDCs treated with MSN**-**MOG**-**Ce expressed lower levels of CD86 and MHC**-**II than LPS**-**treated cells; MSN**-**MOG had no impact on the BMDC activation markers (Fig. 6d and Supplementary Fig. 13). MSN**-**MOG**-**Ce could prevent oxidative stress in BMDCs and maintain their semi-maturity under an inflammatory environment, representing its potential to further aid DCs in enhancing peripheral tolerance in vivo.

Next, to investigate the therapeutic efficacy of ROS**-**scavenging vaccine, EAE**-**induced mice in the late chronic phase were intravenously injected with MSN**-**MOG, MSN**-**MOG**-**Ce, or left untreated. We observed an additional reduction in the clinical score for MSN**-**MOG**-**Ce**-**injected EAE mice compared to that for MSN**-**MOG**-**injected EAE mice (Fig. 6e, see Supplementary Fig. 14 for weight change). After the late therapeutic treatment (day 22), MSN**-**MOG**-**Ce**-**treated EAE mice could take complete coordinated strides, unlike the visibly irregular/wobble walk of MSN**-**MOG**-**treated mice. Intravenous administration of CeNPs or MSN**-**Ce was unable to reduce the disease severity in late chronic phase of EAE (Supplementary Fig. 15a, b). Similarly, the treatment of MSN**-**Ce in disease onset or pegylated CeNPs before disease onset did not prevent EAE progression (Supplementary Fig. 15c–f). We did not observe nanoparticle retention in the CNS of the vaccinated group (Supplementary Fig. 16). These data represent that the therapeutic efficacy of MSN**-**MOG**-**Ce vaccine was attributed to the induction of tolerogenic immune responses rather than direct ROS**-**scavenging in CNS.

To further investigate the immunosuppressive role of ROS**-**scavenging CeNPs in the therapeutic autoimmune disease nanovaccine, we compared the cellular phenotypes and responses in mice with late EAE therapeutics after MSN**-**MOG and MSN**-**MOG**-**Ce administration. The frequency and number of splenic APCs of the treated mice showed no significant change compared to the untreated group, except for an increase in the frequency of B220^+^ cells in the MSN**-**MOG**-**treated group (Supplementary Fig. 17). CD86 expressions on CD11c^+^ DCs, F4/80^+^ macrophages, and B220^+^ cells were significantly lower in the MSN**-**MOG**-**Ce**-**treated group than in the MSN**-**MOG**-**treated group (Fig. 6f). Similarly, CD40 expression on CD11c^+^ DCs and F4/80^+^ macrophages was significantly reduced in the MSN**-**MOG**-**Ce**-**treated group but not in the MSN**-**MOG**-**treated group (Fig. 6g). These results are consistent with the suppressive effects of CeNPs in vitro. There was no difference in MHC**-**II expression on APCs between groups (Supplementary Fig. 18), indicating that loading CeNPs on the nanovaccine only inhibited the expression of costimulatory molecules (CD86 and CD40) without affecting MHC**-**II, thus making the APCs more tolerogenic. Consequently, the MSN**-**MOG**-**Ce**-**treated group exhibited higher Foxp3^+^ Tregs in the spleens of EAE-induced mice (Fig. 6h, i). The percentage of Foxp3^+^ T cells among the CD4^+^ T cells was twice as high in MSN**-**MOG**-**Ce**-**treated mice (day 31) compared to that in untreated mice. The frequency and number of CD4^+^ T cells were similar among the three groups (Supplementary Fig. 19). These results indicate that coating MSN**-**MOG with CeNPs did not elicit APC and helper T-cell proliferation in the spleen but drove the induction of Foxp3^+^ Tregs via the immunosuppressive effects on APCs.

The infiltrated CD4^+^ T cells in CNS is one of the indications to show severity of autoimmune response in EAE. We further examined the infiltration of autoreactive CD4^+^ T cells into the CNS after vaccination. The numbers and percentages of CD4^+^ T cells in the CNS in both MSN**-**MOG**-**treated mice and MSN**-**MOG**-**Ce-treated mice were significantly lower compared to the untreated group (Fig. 6j–l). Importantly, the number of MOG**-**specific CD4^+^ T cells in CNS, which is the main cause of demyelination, was strongly reduced in MSN**-**MOG**-**Ce**-**vaccinated mice (Fig. 6m). The enhanced peripheral tolerance induced by MSN**-**MOG**-**Ce resulted in the inhibition of CNS**-**infiltrating MOG**-**specific CD4^+^ T cells and ameliorated disease severity in the late stage (Fig. 6e). Interestingly, the frequencies of CD11c^+^ and B220^+^ cells in the CNS were greatly suppressed by vaccination (Fig. 6n and Supplementary Fig. 20). A diminishment in MHC**-**II expression on APCs was more significant in the MSN**-**MOG**-**Ce**-**treated group than in MSN**-**MOG counterpart (Fig. 6o and Supplementary Fig. 20), probably owing to the inhibition of CNS**-**infiltrated autoreactive CD4^+^ T cells. Consequently, we observed a reduction in CD4^+^ T-cell frequencies in the cervical lymph nodes of the MSN**-**MOG**-**Ce**-**treated group at the late phase of MS (Fig. 6p and Supplementary Fig. 21). Taken together, the introduction of ROS**-**scavenging nanovaccine did not affect the proliferation of splenic APCs but suppressed their costimulatory signals, which prevents activation of APCs and induce antigen**-**presenting tolerogenic APCs. As a result, a higher frequency of peripheral Tregs could be generated in peripheral lymphoid organ and sequentially inhibit the infiltration of autoreactive CD4^+^ T cells into CNS, which led to suppression of ongoing chronic phase MS.

## Discussion

Delivering self-antigens while maintaining low levels of activation markers on APCs is important for inducing antigen-specific tolerogenic immune responses and Tregs to treat autoimmune diseases^12^. Injecting MOG_35–55_ peptide**-**loaded MSNs intravenously did not significantly upregulate CD86 expression on APCs in the spleen while maintaining expression of MHC**-**II. Furthermore, the introduction of CeNPs on MSN**-**MOG led to intracellular ROS scavenging and further suppressed costimulatory signals on APCs. Importantly, owing to the high loading capacity of MSNs (due to large-sized mesopores) compared to other carriers (Supplementary Fig. 3)^14,20^, an appropriate amount of EAE**-**associated antigen could be delivered to the APCs using a small number of carriers (approximately 87.9 µg MSNs per shot), which prevented undesired APC activation by large numbers of carriers. Consequently, peripheral Tregs were generated in the spleens of animals immunized with MSN**-**MOG.

Peripherally induced Tregs, rather than thymic Tregs, have been proven to effectively prevent the initial priming of encephalitogenic T cells or neuroinflammation^48^. The increase in peripheral Tregs and decrease in pro**-**inflammatory cytokines (IL-17, GM**-**CSF, and TNF**-**α) in the nanovaccine**-**immunized EAE mice were correlated with the reduction in CNS**-**infiltrating CD4^+^ T cells and the amelioration of EAE symptoms, indicating that peripheral tolerance induced by nanoparticle vaccine suppressed the infiltration of autoreactive CD4^+^ T cells into the CNS. Therefore, myelin epitope spreading within the CNS caused by infiltrating effector T cells in EAE could be prevented, which would suppress additional migration of APCs into CNS. This may explain why the percentage of APCs and expression of MHC**-**II on APCs in the CNS of MSN**-**MOG-immunized mice were lower than that of untreated or MSN**-**OVA**-**immunized mice (Fig. 3a, f). It was reported that a fraction of the DC population containing myelin from the CNS can migrate to the cervical lymph nodes and subsequently trigger autoreactive T cells^49^. These autoreactive T cells pass through the disrupted blood–brain barrier to enter CNS, thereby perpetuating the self**-**destruction of the myelin sheath^50^. Therefore, the induction of peripheral Tregs after nanoparticle vaccination suppresses the self**-**destructive circle of MS.

In previous reports, to improve the antigen-specific immune tolerance, autoantigen-encapsulated/conjugated nano/microparticles were additionally loaded with various immunosuppressive molecules including aryl hydrocarbon receptor^13,51,52^, anti**-**inflammatory cytokines^53–55^, immunosuppressive ligands and gene^56–58^, autoantigen**-**loaded MHC^56^, or immunosuppressive small molecules such as rapamycin^15,16^ and vitamin D3^59^. These agents direct DCs and T cells in various immunological pathways to establish immune tolerance^60^. In contrast, we demonstrated in this work that the intrinsic ROS**-**scavenging catalytic property of inorganic CeNPs can suppress APC activation and enhance the tolerogenic properties of DCs even in the presence of pro**-**inflammatory stimuli. To harness its immunosuppressive effect, CeNPs were decorated on MOG**-**loaded MSNs to augment the tolerogenic properties of splenic APCs at late stage of MS (Fig. 6f, g). Consequently, Treg generation was promoted, resulting in efficient therapeutic vaccine efficacy in late chronic phase EAE mice (Fig. 6). It is challenging to restore immune tolerance under the oxidative stress condition of an already disrupted immune system in the chronic phase. Therefore, the ROS**-**scavenging CeNPs play a key role in reducing the intracellular ROS of APCs, effectively keeping the cells at an immature or semi**-**mature phenotype under the inflammatory environment of chronic MS. We believe this is the first report showing a relation between the catalytic properties of a lanthanide element and its immunomodulatory effects triggering Tregs to treat MS.

**Fig. 7:**
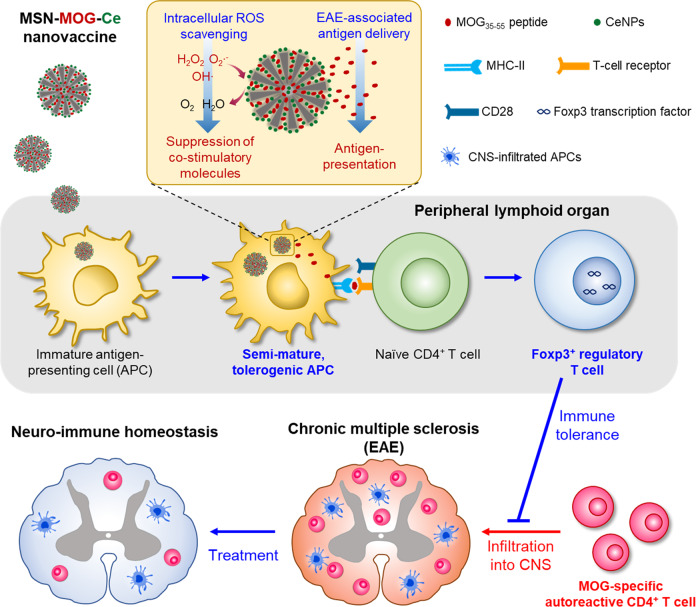
Proposed schematics of the immunosuppressive therapeutic nanoparticle vaccine to treat EAE via re-establishing antigen-specific immune tolerance. The accumulation of CeNPs-decorated, MOG-loaded MSNs in the APCs resulted in the reduction of intracellular ROS and expression of costimulatory molecules (CD86, CD40) on APCs, leading to the suppression of APCs activation, thus making APCs more tolerogenic. The interaction between the MOG peptide presented by MHC-II on semi-mature, tolerogenic APCs and T-cell receptor on naive CD4^+^ T cells enables the induction of Foxp3^+^ Tregs. The peripherally induced Tregs subsequently inhibit the infiltration of MOG-specific autoreactive CD4^+^ T cells to the CNS. Consequently, the reduction of infiltrated CD4^+^ T cells in CNS hampers the neuronal self-destruction and prevents epitope spreading within CNS. Thus, neuro-immune homeostasis can be achieved for the treatment of late, chronic-stage multiple sclerosis.

Taken together, we have designed a simple but effective therapeutic nanovaccine to re**-**establish antigen**-**specific immune tolerance in autoimmune multiple sclerosis (Fig. 7). The nanovaccine platform comprises MSNs carrying EAE**-**associated peptide self**-**antigen and ROS**-**scavenging CeNPs. Systemic delivery of the tolerogenic nanovaccine assisted the generation of peripherally induced Tregs in the spleen, resulting in the reduction of CNS**-**infiltrating T cells and APCs in EAE**-**induced mice. In addition, the ROS**-**scavenging functionality of nanovaccine strongly suppressed APC maturation, increased Treg frequency, and suppressed autoreactive CD4^+^ T cells in CNS, leading to improved therapeutic outcomes with recovered motor function. Our findings show the potential of the ROS**-**scavenging, self**-**antigen**-**carrying porous nanoparticles for engineering immune tolerance against MS and potentially to other autoimmune diseases.

## Methods

### Materials

Hexadecyltrimethylammonium bromide (CTAB), ammonium hydroxide solution, phorbol 12**-**myristate 13**-**acetate (PMA), ionomycin, 6**-**aminohexanoic acid, formic acid, tetraethyl orthosilicate (TEOS), lipopolysaccharide from *Escherichia coli*, rhodamine B isothiocyanate (RITC), and RPMI 1640 were purchased from Sigma**-**Aldrich (St. Louis, MO, USA). Dulbecco’s modified Eagle’s medium (DMEM) was purchased from Lonza (Basel, Switzerland). 2′,7**-**Dichlorofluorescein diacetate (H_2_DCFDA) was purchased from Invitrogen (Carlsbad, CA, USA). Cerium (III) nitrate hexahydrate was purchased from Alfa Aesar (Tewksbury, MA, USA). Ethanol, methanol, hydrochloric acid, and ethyl acetate were purchased from Samchun (Seoul, South Korea). Endotoxin**-**free ultra-pure water was purchased from EMD Millipore (MA, USA). MOG_35–55_ peptide (sequence: MEVGWYRSPFSRVVHLYRNGK) and OVA_323–339_ peptide (sequence: ISQAVHAAHAEINEAGR) were synthesized by Anygen (Gwangju, South Korea). Methoxy poly(ethylene glycol) succinimidyl glutarate (M_w_ = 5000) was purchased from SunBio (Gyeonggi, South Korea). Fluorescein isothiocyanate (FITC)**-**conjugated anti**-**CD3, eFluor 450 and R**-**phycoerythrin (PE)**-**conjugated anti**-**F4/80, PE**-**Cyanine7 (PE**-**Cy7)**-**conjugated anti**-**CD4, APC**-**conjugated anti**-**CD11c, eFluor 450**-**conjugated anti**-**CD86, FITC**-**conjugated anti**-**CD40, PE**-**Cy7**-**conjugated anti**-**B220, FITC**-**conjugated anti**-**MHC**-**II, and APC**-**conjugated anti**-**Foxp3 monoclonal antibodies were purchased from eBioscience (CA, USA). PE**-**conjugated I**-**A^b^ MOG_35–55_ tetramer was purchased from MBL (Japan). FcR blocking reagent, APC**-**conjugated anti**-**Foxp3, PE**-**conjugated anti**-**CD25, FITC**-**conjugated anti**-**IFN**-**γ, PE**-**Vio770**-**conjugated anti**-**CD4, APC**-**conjugated anti**-**IL**-**10, PE**-**conjugated anti-IL-17A monoclonal antibodies, CD4^+^ T**-**cell isolation kit, LS column, and MidiMACS separator were purchased from Miltenyi Biotec (Bergisch Gladbach, Germany). Pacific blue**-**conjugated anti**-**mouse TCR Vα2 antibody was purchased from BioLegend (CA, USA). Alexa Fluor 647**-**conjugated anti**-**Iba1/AIF**-**1 monoclonal antibody was purchased from Cell Signaling Technology, Inc.

### Synthesis of large-pore mesoporous silica nanoparticles

MSNs were synthesized according to a previous study^23^. First, 500 µL of Fe_3_O_4_ (6 mg/mL) nanocrystals, which were prepared from an iron**-**oleate complex by a heat**-**up reaction, was mixed with 10 mL of a 0.055 M CTAB aqueous solution with vigorous stirring for 30 min. The mixture was then heated at 60 °C for 15 min before being poured into 95 mL of deionized (DI) water. Subsequently, 3 mL of ammonium hydroxide solution, 5 mL of methanol, 20 mL of ethyl acetate, and 500 µL of TEOS were added to the mixture and allowed to react overnight. The resulting nanoparticles were washed thrice with ethanol. The CTAB template and Fe_3_O_4_ nanocrystal core were removed by stirring the MSNs in ethanol containing HCl for 3 h at 60 °C. Finally, the MSNs were washed thrice with ethanol and stored in ethanol until use. To track MSN accumulation in immune cells in vivo, RITC**-**MSN was prepared by mixing RITC with MSNs in methanol for 48 h under dark conditions. An intensive wash was applied to completely remove the unbound RITC.

### Synthesis of cerium oxide nanoparticles

6**-**Aminohexanoic acid (6**-**AHA) (10 mmol) and cerium (III) nitrate hexahydrate (2.5 mmol) were dissolved in 60 and 50 mL of DI, respectively. The 6**-**AHA solution was then heated. When the temperature of the solution reached 95 °C under continuous stirring, 70 µL of HCl was added. Thereafter, the cerium (III) salt solution was immediately poured into the heated 6**-**AHA solution under vigorous stirring. To produce CeNPs with a 3 nm diameter, the mixture was allowed to react for 1 min before washing thrice with excess acetone. The CeNPs were collected under vacuum pressure and re**-**dispersed in sterile DI water. To pegylate CeNPs, 10 mg CeNPs were allowed to react with 250 mg methoxy poly(ethylene glycol) succinimidyl glutarate in 20 mL ethanol at pH 8. The resulted nanoparticles were washed thrice with excess acetone and collected after being dried under vacuum pressure.

### MSN and CeNP characterization

The porous properties of MSNs were measured using the Brunauer**–**Emmett–Teller (BET) method. The nanoparticle size and morphology were analyzed using transmission electron microscopy (JEM-2100F, JEOL, Akishima, Japan) and scanning electron microscopy (JSM**-**7000F, JEOL), respectively. Energy dispersive X**-**ray spectroscopy elemental mapping was conducted using a JEM**-**2100F field emission electron microscope (JEOL, Akishima, Japan). The concentration of cerium was measured using inductively coupled plasma-optical emission spectrometry (ICP**-**OES, Varian, CA, USA).

### Cell Counting Kit (CCK)-8 cytotoxicity assay

10^4^ RAW 264.7 cells (ATCC) were seeded per well in a 96**-**well plate and incubated for 24 h at 37 °C. Then, various concentrations of MSNs (25, 50, 100, and 200 µg/mL) and cerium (5, 10, 20, 50, and 100 µg/mL) were incubated with the cells for the next 24 h. Finally, 10 µL of CCK**-**8 solution (Dojindo, Japan) was added to each well and incubated for 2 h at 37 °C. Absorbance was measured at 450 and 600 nm using a microplate reader (Thermo Fisher Scientific, MA, USA). The cell viability was calculated according to the manufacturer’s instructions.

### Preparation of peptide-loaded MSNs

Five hundred microliters of MOG_35–55_ peptide solution (1 µg/µL in DI water) and five hundred microliters OVA_323–339_ peptide solution (1 µg/µL in DI water) were separately mixed with 1 mg MSNs each, and incubated for 3 h at 25 °C. Subsequently, the nanoparticles were washed in DI water three times under sterilized conditions, and the loading efficiency was measured using the Pierce BCA Protein Assay Kit (Thermo Fisher, MA, USA). The peptide-loaded nanoparticles were finally re**-**dispersed in saline buffer prior to retro-orbital injection using a BD insulin syringe (BD, NJ, USA). To prepare MSN**-**MOG-Ce, MOG_35–55_**-**loaded MSNs were mixed with 1 mg CeNPs (2 mg/mL) in DI water and gently shaken for 5 min. The mixture was then centrifuged (10,000 × g, 5 min) and washed twice with DI water to remove unbound CeNPs. The loaded CeNPs were measured using the ultraviolet–visible method at 310 nm. Finally, the nano**-**composition was re**-**dispersed in saline buffer prior to retro**-**orbital injection using a BD Insulin Syringe.

### Animals

Female C57BL/6 mice aged 9 weeks were purchased from OrientBio (Seongnam, South Korea). The experimental and control animals were co-housed under specific pathogen**-**free condition during the study. Female OT**-**II (C57BL/6**-**Tg(TcraTcrb)425Cbn/Crl) mice of 7**–**9-week age were used for in vitro generation of Tregs. OT**-**II mice were a kind gift from Prof. Suk-Jo Kang from Korea Advanced Institute of Science and Technology (KAIST). Animals were acclimatized for at least 1 week before immunization. At the end of each study, mice were euthanized by carbon dioxide (flow rate: 3 L/min). All experiments were approved by the Institutional Animal Care and Use Committee of Sungkyunkwan University (SKKUIACUC, No. 2020**-**01**-**15**-**1).

### In vivo cellular uptake in spleen

Twenty-four hours prior to intravenous administration of RITC**-**MSN into C57BL/6 mice, splenocytes were isolated. The cells were stained with FcR blocking reagent for 10 min at 4 °C and washed. Subsequently, cells were stained with antibodies against CD11c, F4/80, B220, and CD3 for 20 min at 4 °C. Positive signals from stained surface markers were gated among the RITC^+^ cells to determine the cell types that engulfed RITC**-**MSN.

### EAE induction

EAE was induced in female C57BL/6 mice aged 10~11 weeks using a kit (EK**-**2110) from Hooke Laboratory (Lawrence, MA, USA). Briefly, on day 0, mice were subcutaneously injected with an emulsion containing MOG_35–55_ peptide and complete Freund’s adjuvant (CFA), in the lower and upper back. After 2 and 24 h, the animals were intraperitoneally injected with pertussis toxin (PTX) according to the manufacturer’s instructions. EAE clinical score was evaluated after day 8, post**-**EAE induction based on the standard protocol in a blinded manner (0, no obvious symptoms; 0.5, tip of tail was limp; 1, limp tail; 1.5, limp tail and hind leg inhibition; 2, limp tail and weakness of hind legs; 2.5, limp tail and dragging of hind legs; 3, complete paralysis of hind legs; 3.5, complete paralysis of hind legs and hind legs together on one side of the body; 4, full hind leg and partial front leg paralysis; 4.5 full hind leg and partial front leg paralysis, no movement). Paralyzed mice were given easier access to water and food. Mice were euthanized if any of the following conditions were observed unable to eat, unresponsive when scored as 4, when scored as 4 for two consecutive days, both hind limbs and forelimbs were completely paralyzed.

### Therapeutic vaccine studies

For semi-therapeutic treatment (vaccination after disease establishment but before the onset of symptoms), the mice were injected with different formulations of material components (MSN, MOG, MSN**-**MOG, MSN**-**OVA) on days 4, 7, and 10 after EAE induction or left untreated. Supplementary Fig. 3 shows the dose for each formulation in detail. To examine the immune responses after semi**-**therapeutic treatment of MSN**-**OVA, MSN**-**MOG, or no treatment in EAE**-**induced mice, mice from each group were euthanized for spleen and spinal cord collection on day 10 after the final injection (the respective treatments were administered on days 4, 7, and 10 post**-**EAE induction). For the early therapeutic study, EAE**-**induced mice were injected with MSN-MOG or left untreated on days 12, 15, and 18. For the late therapeutic study, EAE**-**induced mice were injected with MSN-MOG or left untreated on days 15, 18, and 21.

### In vivo CD4^+^CD25^+^Treg depletion

EAE mice were intraperitoneally injected with anti**-**CD25 antibody (BioXCell, clone: PC**-**61.5.3) at a dose 400 µg/mouse on days 5, 9, and 13 after EAE induction. Equal dose of rat IgG1 anti**-**horseradish peroxidase isotype control (BioXCell, clone: HRPN) was used as control. Approximately 90% of CD4^+^CD25^+^ T cells were depleted in peripheral blood, analyzing by flow cytometry 2 days after the last antibody injection. The MSN**-**MOG vaccine was intravenously injected into the EAE mice on days 4, 7, and 10 after EAE induction.

### Histology

The vertebral columns were collected on day 50 post**-**EAE induction and fixed in 4% buffered formaldehyde solution for 48 h. The tissues were then washed in DI water before decalcification in an aqueous solution containing 4% formic acid and 4% hydrochloric acid for 72 h. The acid solution was replaced daily. Subsequently, the mouse spines were neutralized in an ammonia solution, washed in DI water, and embedded in paraffin. The tissues were cut into 4-µm thick sections. Finally, the spinal cord sections were stained with H&E, and an optical microscope (ECLIPSE Ti−U, Nikon, Japan) was used to visualize them.

### Tissue processing

Spleens were excised and processed by mechanical disruption using a 70 µm cell strainer. Cells were then centrifuged for 5 min, 400 × g, 4 °C and treated with ammonium-chloride-potassium (ACK) lysing buffer (Lonza) for 4 min to remove red blood cells. Splenocytes were then filtered through a 40 µm cell strainer and washed in cold PBS. Spinal cords were dissociated in PBS containing 1 mg/mL collagenase type IV and 20% EDTA/trypsin and incubated at 37 °C for 20 min. RPMI 1640 containing 10% fetal bovine serum was added to each sample to inhibit enzymatic activity, and the cells were filtered through a 40 µm cell strainer. The cells were collected by centrifugation according to standard protocol.

### Flow cytometry

Immediately after stimulation or obtaining single**-**cell suspensions from tissues, cells were incubated with the FcR blocking reagent for 10 min at 4 °C to prevent non-specific binding. Then, antibodies against surface markers CD11c, B220, F4/80, CD86, CD40, MHC**-**II were used to stain for APC analysis. For T**-**cell analysis, I**-**A^b^ MOG_35–55_ tetramer was used to stain the cells for 40 min at 4 °C, then antibodies against CD3 and CD4 were used to stain the cells for 20 min at 4 °C before being washed in FACS buffer. After that, the cells were either analyzed immediately or fixed and permeabilized for transcription factor staining. Foxp3/Transcription Factor Staining buffer set (eBioscience 00**-**5523**-**00, CA, USA) was used to fix and permeabilize the cells prior to staining with antibody against Foxp3. Suitable isotype control antibodies were used as the negative controls. For the analysis of Iba1 expression in CNS cells, the cells were first fixed and permeabilized by Intracellular Fixation & Permeabilization buffer set (eBioscience 88**-**8824**-**00, CA, USA) prior to Iba1 staining for 1 h. The stained cells were analyzed using a MACSQuant VYB flow cytometer (Miltenyi Biotec, Bergisch Gladbach, Germany). All cells were gated based on forward-scatter and side-scatter characteristics to exclude dead cells and debris. Thereafter, the forward-scatter height (FSC**-**H) and forward-scatter area (FSC**-**A) parameters were used to determine the single-cell population. Finally, the frequency of positively stained cells for each marker was recorded based on the isotype control antibodies. Examples of the gating strategies are shown in Supplementary Fig. 22. Data were analyzed using FlowJo X 10.0 (Becton, Dickinson and Company).

### Cytokine recall study

Half the mice from each group were euthanized for splenocyte collection and on day 3, and other half were euthanized for splenocyte collection on day 10 after the final injection. Then, splenocytes (1 × 10^6^) isolated from each mouse were restimulated with 20 µg/mL of MOG_35–55_ peptide for 72 h. The culture supernatant was collected and stored at −80 °C until use. The secreted cytokines IL-10, GM**-**CSF, TNF**-**α, and IL-17A were quantified by enzyme-linked immunosorbent assay (ELISA, R&D Systems, Minneapolis, MN, USA) according to the manufacturer’s instructions.

### BMDC culture

Bone marrow cells from the femurs of C57BL/6 mice were isolated and filtered using a 70 µm cell strainer. The cells were then cultured in complete RPMI 1640 supplemented with 10% heat-inactivated fetal bovine serum, 1% penicillin/streptomycin (Sigma**-**Aldrich), 50 µM β**-**mercaptoethanol (Sigma-Aldrich), and 20 ng/mL GM**-**CSF (PeproTech, NJ, USA). The culture medium was refreshed on days 3 and 6. Differentiated cells from days 7 to 9 were used for the cell activation study.

### CD4^+^ T cell isolation and culture

Spleen and lymph nodes of OT**-**II mice were first processed to obtain single**-**cell suspensions. Purified CD4^+^ T cells were isolated by magnetic activated cell sorting (MACS) using CD4^+^ T cell isolation kit (Miltenyi Biotec, 130**-**104**-**454). OT-II CD4^+^ T cells were then washed and cultured with BMDCs in complete RMPI 1640 supplemented with 10% heat-inactivated fetal bovine serum (Gibco), 1% penicillin/streptomycin (Sigma**-**Aldrich), 50 µM β**-**mercaptoethanol (Sigma**-**Aldrich) at 37 °C in 5% CO_2_.

### Apoptosis assay

BMDCs were seeded in 6**-**well culture plates (1 × 10^6^ cells/well). The cells were then co**-**incubated for 48 h with pegylated CeNPs at different cerium concentrations. Subsequently, the cells were washed with FACS buffer and stained using the FITC Annexin V Apoptosis Kit with PI (BioLegend, CA, USA) according to the manufacturer’s instructions before performing flow cytometry analysis.

### BMDC activation study

BMDCs were seeded in 6**-**well culture plates (1 × 10^6^ cells/well). The cells were then treated with either pegylated CeNPs (Ce, 50 µM cerium), OVA_323–339_ peptide (OVA, 1 µg/mL), pegylated CeNPs and OVA_323–339_ peptide (Ce + OVA), MSN**-**MOG, MSN**-**MOG**-**Ce, or left untreated for 24 h, followed by stimulation with 1 µg/mL or 100 ng/mL LPS for the next 24 or 12 h. Finally, the cells were washed and stained with H_2_DCFDA and antibodies against CD11c, CD86, CD40, and MHC**-**II before flow cytometry analysis.

### T-cell differentiation in vitro

Day 7 BMDCs were treated with PBS (control), pegylated CeNPs (Ce, 50 µM cerium), OVA_323–339_ peptide (OVA, 1 µg/mL), or pegylated CeNPs plus OVA (Ce + OVA) in 24 h, following by LPS treatment (1 µg/mL) in the next 24 h. Then BMDCs were washed twice and co**-**cultured with OT**-**II CD4^+^ T cells at 1:10 (30,000:300,000) BMDCs to T-cell ratio for 72 h. Subsequently, the cells were stimulated in a culture medium containing PMA (100 ng/mL), ionomycin (1 µg/mL) for 5 h and protein transport inhibitor (GolgiStop, BD Bioscience) in the last 3 h. Finally, cells were fixed, permeabilized, and stained with antibodies against Vα2, CD4, CD25, Foxp3, IL**-**10, IFN**-**γ, IL-17A before flow cytometry analysis.

### ROS-scavenging study

BMDCs were seeded in 6**-**well culture plates (1 × 10^6^ cells/well). The cells were then treated with either MSN**-**MOG or MSN**-**MOG**-**Ce, or left untreated for 24 h, followed by stimulation with 100 ng/mL LPS to induce excessive intracellular ROS for the next 12 h. Subsequently, the cells were stained with 5 µM H_2_DCFDA and CD11c antibody for 20 min at 4 °C before being washed and analyzed by flow cytometry.

### Statistics and reproducibility

EAE clinical score values were expressed as the mean ± standard error (SE). All other values are expressed as mean ± standard deviation (SD) unless indicated otherwise. An unpaired two**-**tailed *t***-**test was performed to compare the statistical significance between the two groups. For multiple comparisons, one**-**way ANOVA was performed using GraphPad Prism 7.00; the types of multiple comparison tests are provided in figure captions. In all cases, difference between groups was considered significant at *P* < 0.05. GraphPad Prism 7.00, Microsoft Excel 2013, and Microsoft PowerPoint 2013 for Windows were used to draw graphs and schemes. No data were excluded from the analyses. Mice from the same litter were induced with EAE and randomly distributed in cages prior to vaccination. Randomization was conducted in semi**-**therapeutic treatment of EAE mice. For early and late therapeutic studies, only successful EAE**-**induced mice were used at the time of vaccination. EAE**-**free curves were calculated using a log**-**rank (Mantel–Cox) test. No statistical method was used to predetermine the sample size. All animal studies were monitored by two investigators and the investigators were blinded to group allocation during the data collection.

### Reporting summary

Further information on research design is available in the Nature Portfolio Reporting Summary linked to this article.

## Supplementary information

Supplementary Information

Description of Additional Supplementary Files

Supplementary Movie 1

Reporting Summary

## Data Availability

The data necessary to reproduce the graphs presented within this article are provided in the Source Data file. Supplementary Movie 1 is provided in the Supplementary Information file. Source Data are provided with this paper.

## Source data

Source Data
