# Supplementary material for: Immunosuppressive biomaterial-based therapeutic vaccine to treat multiple sclerosis via re-establishing immune tolerance
Source: Nat Commun. 2022 Dec 2;13:7449. doi: 10.1038/s41467-022-35263-9 (PMC9718828; doi:10.1038/s41467-022-35263-9)
Supplement: Supplementary file 1 — Supplementary Information [file 41467_2022_35263_MOESM1_ESM.pdf]

Supplementary Information

for

Immunosuppressive Biomaterial-based Therapeutic Vaccine to Treat Multiple Sclerosis via Re-establishing Immune Tolerance

Thanh Loc Nguyen[1], *Youngjin Choi*[1,2], *Jihye Im*[1], *Hyunsu Shin*[1], *Ngoc Man Phan*[1], *Min Kyung Kim*[3], *Seung Woo Choi*[3], *and Jaeyun Kim*[1,3,4,5,*]

[1] School of Chemical Engineering, Sungkyunkwan University (SKKU), Suwon 16419, Republic of Korea

[2] Center for Theragnosis, Biomedical Research Institute, Korea Institute of Science and Technology (KIST), Seoul 02792, Republic of Korea

[3] Department of Health Sciences and Technology, Samsung Advanced Institute for Health Sciences & Technology (SAIHST), Sungkyunkwan University (SKKU), Seoul 06355, Republic of Korea

[4] Biomedical Institute for Convergence at SKKU (BICS), Suwon 16419, Republic of Korea

[5] Institute of Quantum Biophysics (IQB), Sungkyunkwan University (SKKU), Suwon 16419, Republic of Korea

* Correspondence should be addressed to Jaeyun Kim, email: kimjaeyun@skku.edu

Keywords: multiple sclerosis, immune tolerance, cerium oxide nanoparticles, mesoporous silica, immunotherapy

Supplementary Figures

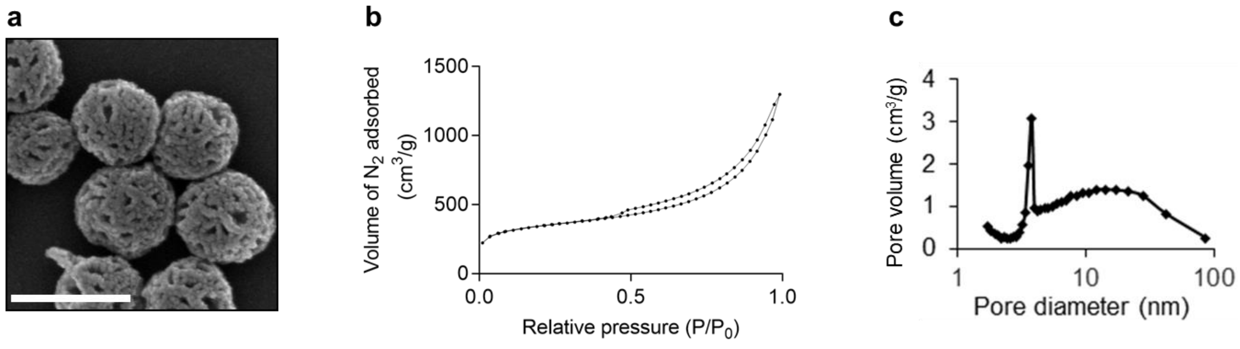

Supplementary Figure 1. MSN characterization (**a**) Scanning electron microscope (SEM) image of MSNs, scale bar: 200 nm; the experiment was repeated independently at least three times. (**b**) Nitrogen adsorption/desorption isotherms of MSNs, P/P_0 indicates the ratio between equilibrium (P) and saturation (P_0) pressure of nitrogen at the adsorption temperature. (**c**) The distribution of desorption pore-size of MSNs.

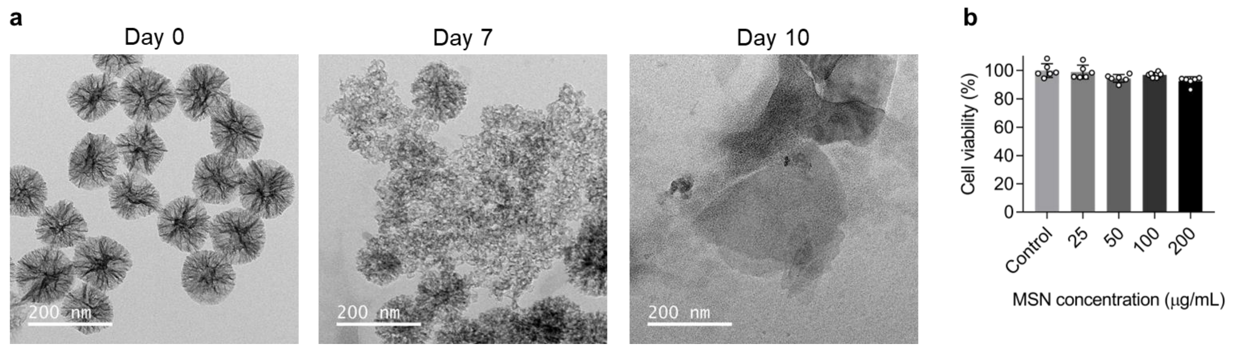

Supplementary Figure 2. In vitro degradation and cell toxicity of MSNs. (**a**) TEM images of MSNs incubated in PBS at 37 ℃ over time. (**b**) Cell Counting Kit (CCK)-8 viability assay of RAW 264.7 cells incubated for 24 h with various concentrations of MSNs (n = 6 biologically independent samples). Data in (**b**) are represented as mean ± standard deviation (SD).

	MSN-MOG (1xMSN-MOG)	MSN-OVA
Loaded peptide (μg) per mg MSNs	113.80 ± 2.08	87.49 ± 15.44
Amount of peptide per injection (μg)	10 ± 0.18	10 ± 1.76
Amount of MSN per injection (μg)	87.87	114.30

Supplementary Figure 3. The amounts of loaded MOG peptide and OVA peptide per 1 mg MSNs and the administration doses of each peptide and MSNs used in this study.

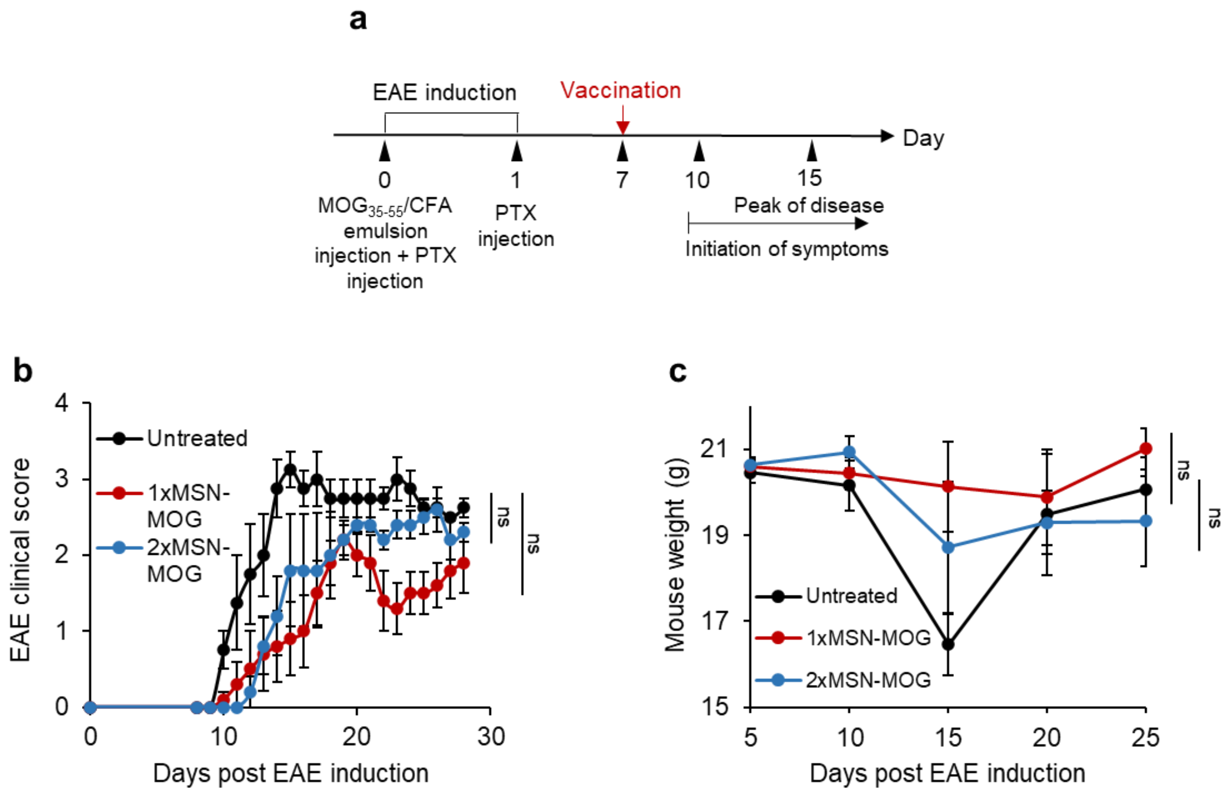

Supplementary Figure 4. The effect of MSN amount on semi-therapeutic efficacy (**a**) EAE was induced in C57BL/6 mice before intravenous injection with different amounts of MSNs on day 7. The amount of MOG_{35-55} loaded was unchanged. (**b**, **c**) EAE clinical scores and body weights, of mice treated with normal (1× MSN-MOG) and double amount of MSNs (2× MSN-MOG), while maintaining the same dose of the MOG peptide. n = 4 (untreated) or 5 (1× MSN-MOG and 2× MSN-MOG) biologically independent animals. Data in (**b**, **c**) are represented as mean ± standard error (SE) and were subjected to one-way ANOVA. Dunnett's multiple comparisons tests were performed in (**b**, **c**), ns = not significant.

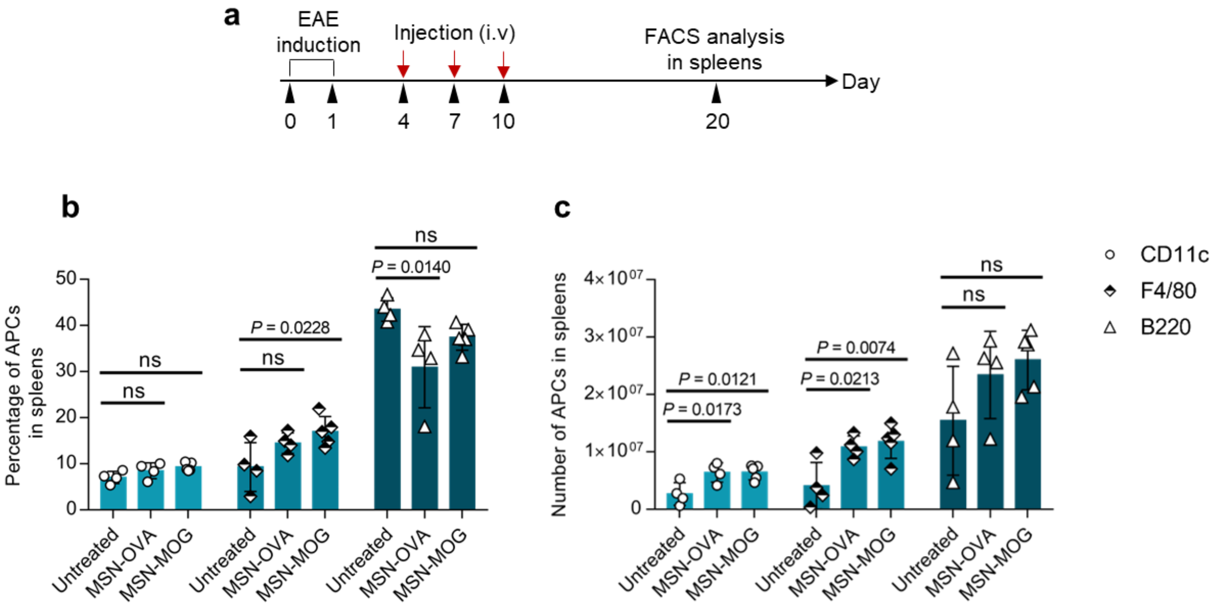

Supplementary Figure 5. Analysis of APCs in spleens after administering semi-therapeutics (**a**) EAE was induced in C57BL/6 mice on days 0 and 1, which was followed by intravenous injection of MSN-OVA, MSN-MOG, or no treatment. Splenocytes were isolated on day 20 after EAE induction for flow cytometry analysis. (**b**, **c**) The percentages and numbers of APCs, namely, $CD11c^+$, $F4/80^+$, and $B220^+$ cells, respectively. n = 4 (untreated and MSN-OVA) or 5 (MSN-MOG) biologically independent animals. The data in (**b**, **c**) are represented as mean ± SD and were subjected to a one-way ANOVA. Dunnett's multiple comparisons tests were performed in (**b**, **c**). $P < 0.05$ was considered significant, ns = not significant.

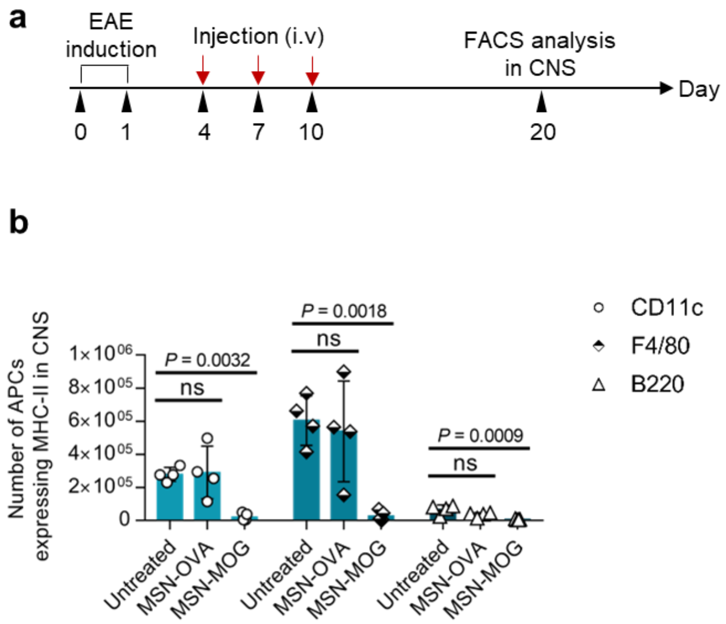

Supplementary Figure 6. Analysis of APCs in the CNS after administering semi-therapeutics (**a**) EAE was induced in C57BL/6 mice on days 0 and 1, which was followed by intravenous injection of MSN-OVA, MSN-MOG, or no treatment. Cells were isolated from the spinal cord on day 20 after EAE induction for flow cytometry analysis. (**b**) The numbers of MHC-II molecules expressed by APCs, namely, $CD11c^+$, $F4/80^+$, and $B220^+$ cells. n = 4 (untreated and MSN-OVA) or 5 (MSN-MOG) biologically independent animals. The data in (**b**) are represented as mean ± SD and were subjected to a one-way ANOVA. Dunnett's multiple comparisons test was performed in (**b**). $P < 0.05$ was considered significant, ns = not significant.

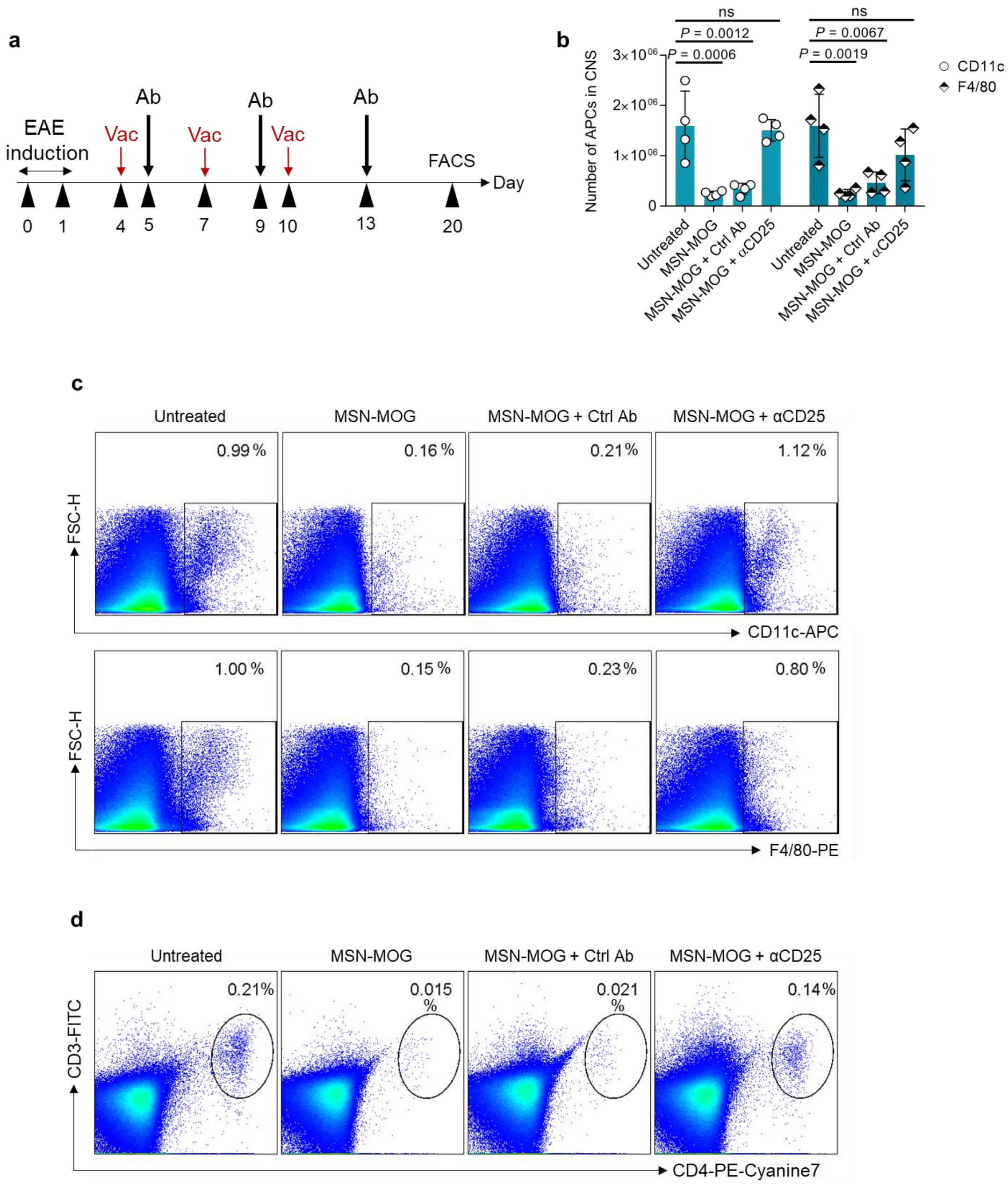

Supplementary Figure 7. Tregs are indispensable for the establishment of immune tolerance by MSN-MOG in EAE mice (**a**) EAE mice were injected with MSN-MOG, MSN-MOG and control antibody (MSN-MOG + Ctrl Ab), MSN-MOG and anti-CD25 antibody (MSN-MOG + αCD25), or left untreated. (**b**) Number of APCs in the spinal cord on day 20, n = 4 biologically independent animals. (**c**, **d**) representative plots showing the percentage of APCs (CD11c^{+} cells and F4/80^{+} cells) and CD4^{+} T-cells in the spinal cord on day 20, respectively. Data in (**b**) are represented as mean ± SD and were subjected to a one-way ANOVA with Dunnett's multiple comparisons tests. P < 0.05 was considered significant, ns = not significant.

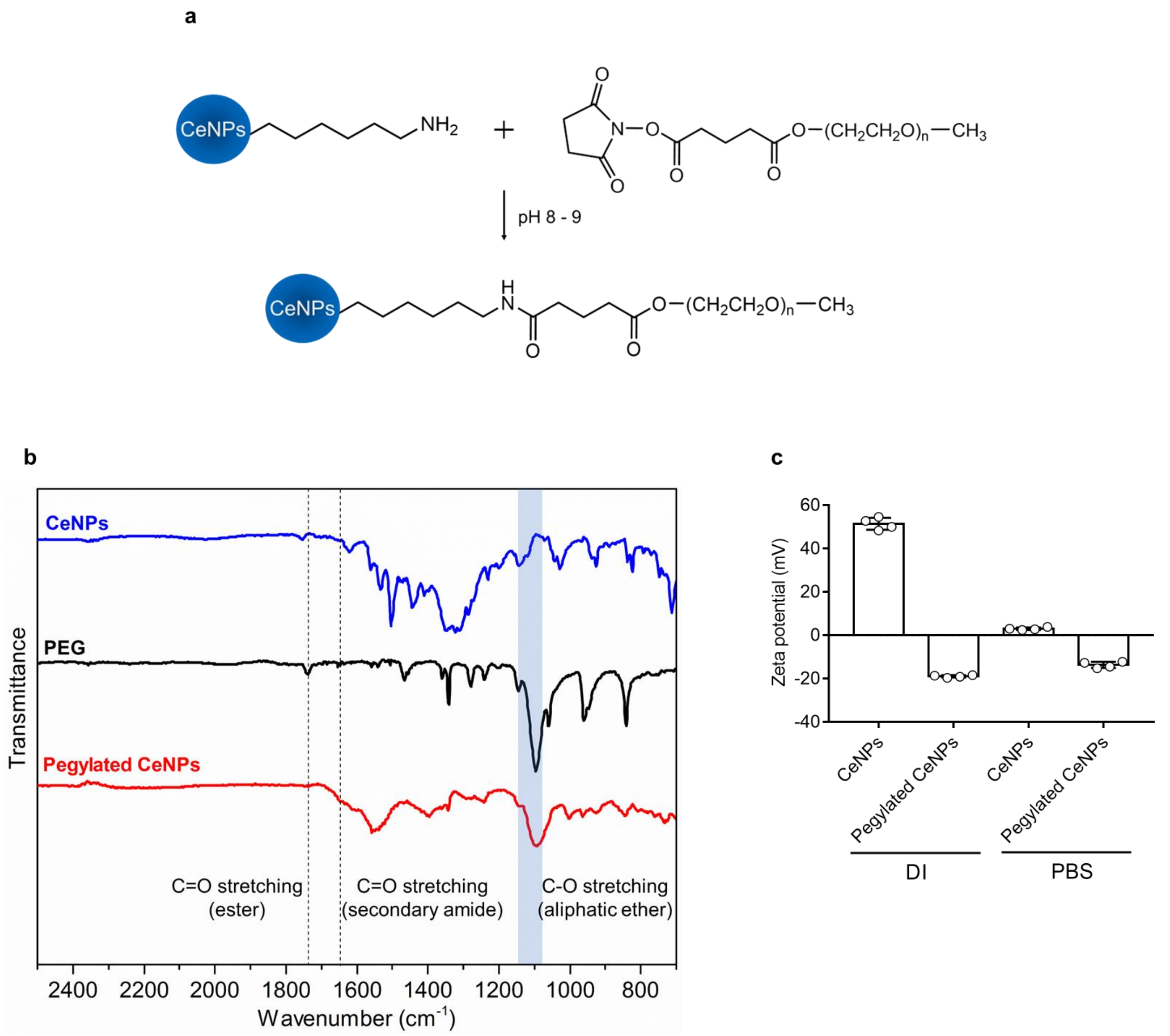

Supplementary Figure 8. PEGylation of cerium oxide nanoparticles (**a**) Schematic demonstration of the surface functionalization of cerium oxide nanoparticles by methoxy poly(ethylene glycol) succinimidyl glutarate (PEG). (**b**) FTIR spectra of CeNPs, PEG, and Pegylated CeNPs. (**c**) Zeta potential of CeNPs and pegylated CeNPs in DI water and PBS, n = 4 biologically independent samples. Data in (**c**) are represented as mean ± SD.

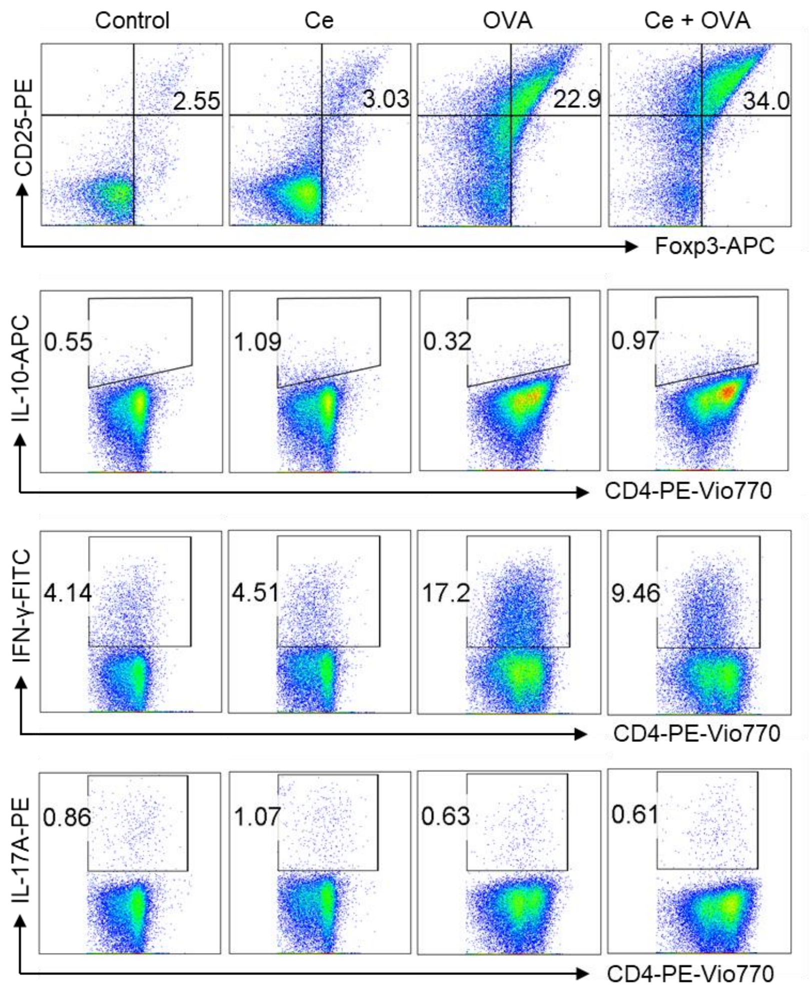

Supplementary Figure 9. CeNPs induced Tregs in vitro BMDCs were incubated with pegylated CeNPs (Ce, 50 µM cerium), $OVA_{323\text{-}339}$ (OVA, 1 µg/mL), pegylated CeNPs plus $OVA_{323\text{-}339}$ (Ce + OVA), or left untreated for 24 h; following by LPS treatment (1 µg/mL) for the next 24 h before being co-cultured with OT-II $CD4^+$ T-cells for 72 h. Figure shows the representative pseudocolor plots of the expression of $CD25^{high}Foxp3^+$, IL-10, IFN-γ, and IL-17A in the gate of OT-II $CD4^+$ T-cells.

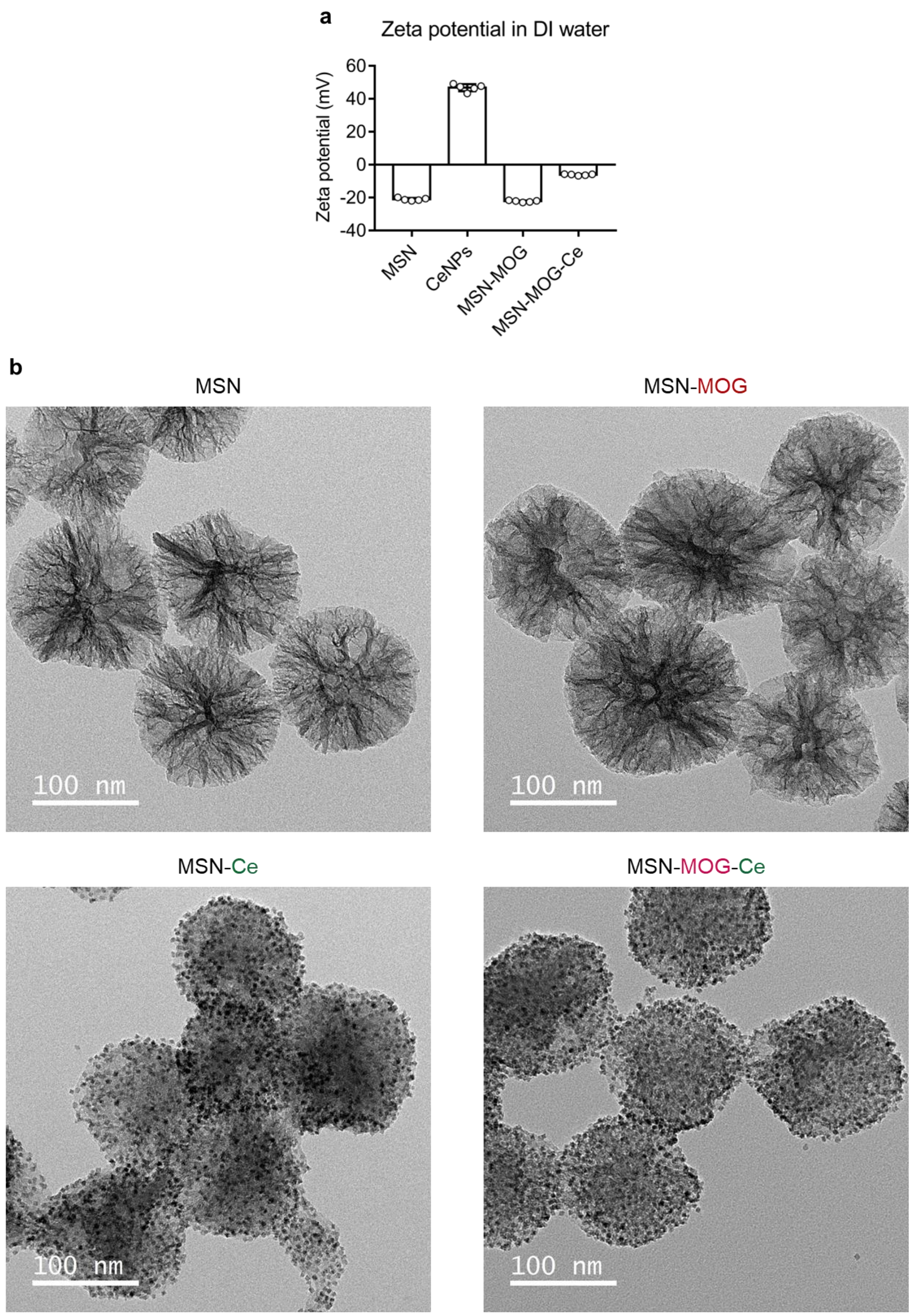

Supplementary Figure 10. Characterization of nanovaccine (**a**) Zeta potential of MSN-MOG-Ce vaccine and its constituents in DI water, n = 5 independent samples. (**b**) Representative TEM images of MSN, MSN-MOG, MSN-Ce, and MSN-MOG-Ce. The data in (**a**) are represented as mean ± SD.

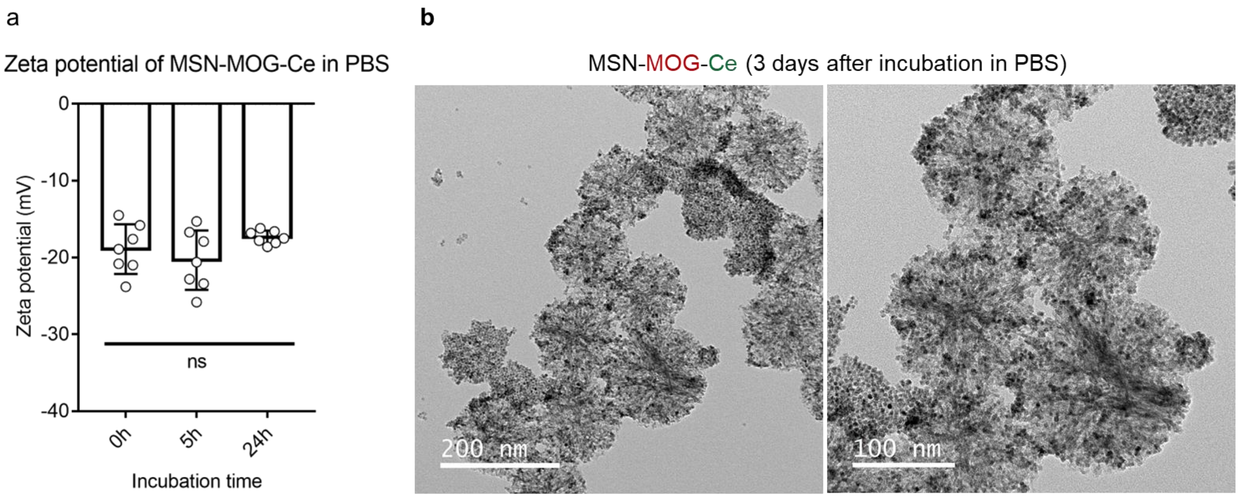

Supplementary Figure 11. Stability of MSN-MOG-Ce nanoparticles in PBS. (**a**) Zeta potential of MSN-MOG-Ce at different time points in PBS after formulating, n = 7 independent samples. (**b**) TEM images of MSN-MOG-Ce 3 days in PBS at 37 °C. The data in (**a**) are represented as mean ± SD and were subjected to a one-way ANOVA, ns = not significant.

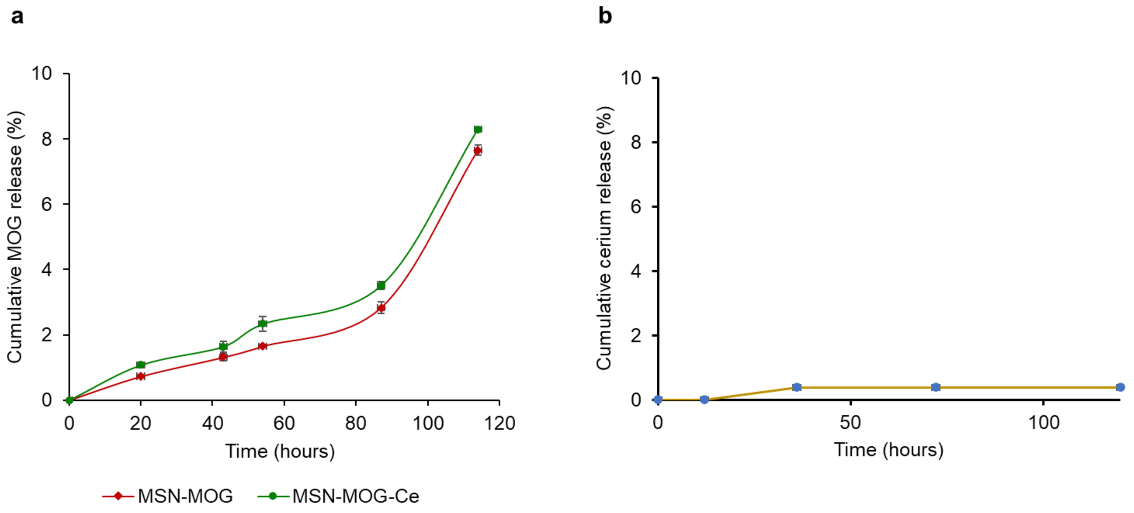

Supplementary Figure 12. The release of MOG and CeNPs. (**a**) The cumulative release of MOG from MSN-MOG and MSN-MOG-Ce, n = 3 independent samples. (**b**) The release of cerium from MSN-MOG-Ce, n = 2 independent samples. The data are represented as mean ± SD.

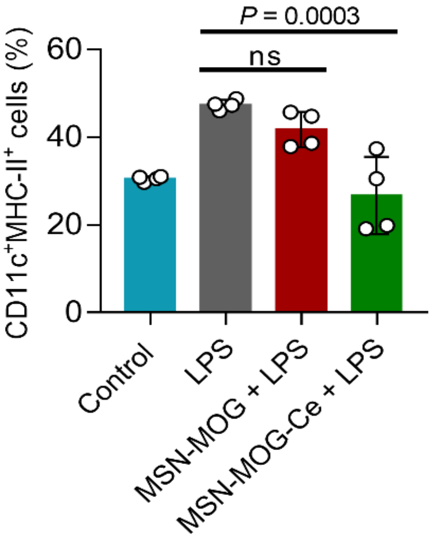

Supplementary Figure 13. In vitro BMDCs suppression. BMDCs were treated with MSN-MOG, MSN-MOG-Ce, or left untreated for 24 h, followed by stimulation with 100 ng/mL LPS for the next 12 h; n = 4 biologically independent samples. Figure shows the percentage of $CD11c^{+}$ BMDCs expressing MHC-II. The data are represented as mean ± SD and were analyzed by one-way ANOVA with Tukey's multiple comparisons test, ns = not significant.

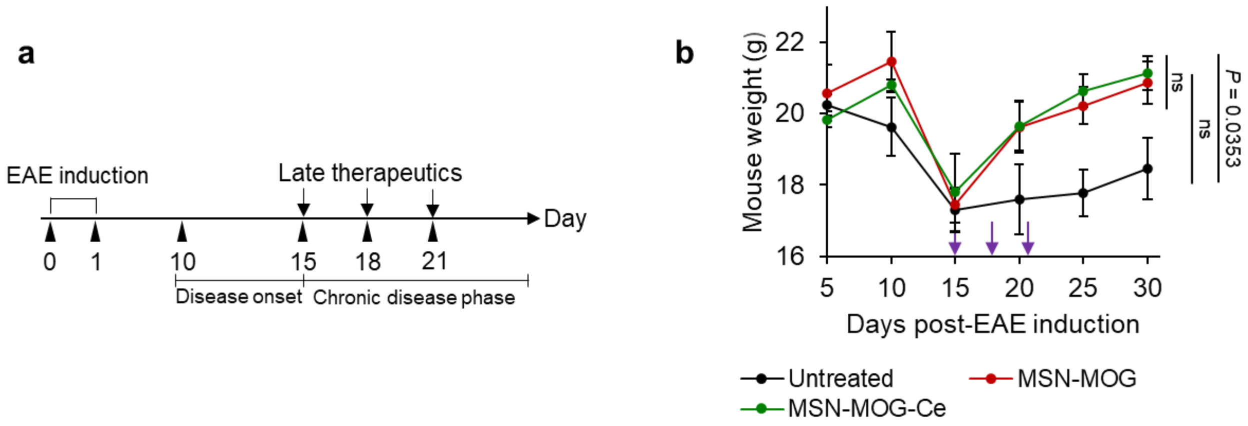

Supplementary Figure 14. Late therapeutic treatment (**a**) EAE was induced in C57BL/6 mice, followed by thrice intravenous injection of MSN-MOG or MSN-MOG-Ce, or left untreated, starting from day 15 (late therapeutics); n = 5 biologically independent animals. (**b**) Body weights of mice during the study; the arrows indicate the injection time points. The data in (**b**) are represented as mean ± SE and were subjected to a one-way ANOVA with Tukey's multiple comparisons test. $P < 0.05$ was considered significant, ns = not significant.

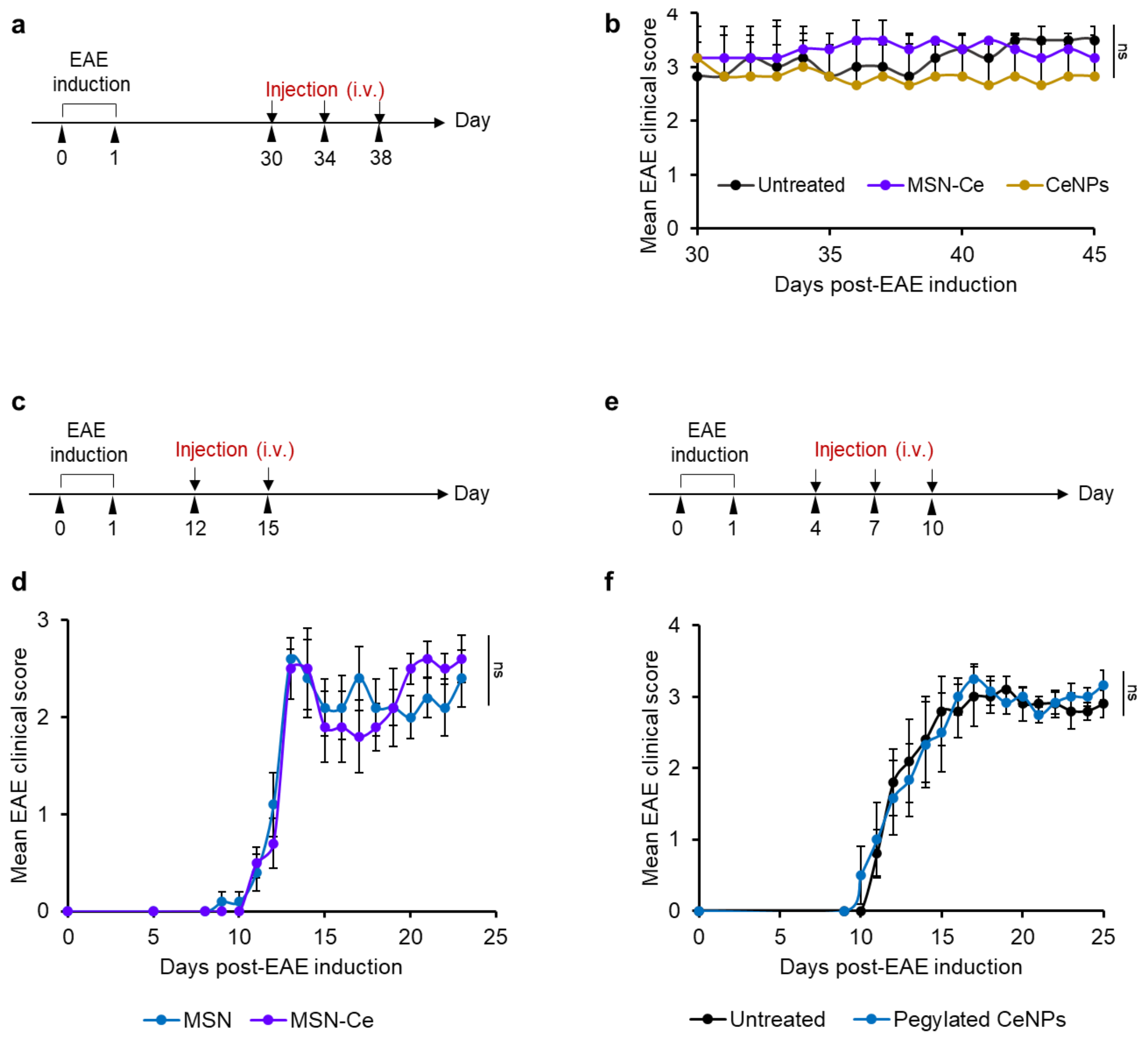

Supplementary Figure 15. Cerium oxide nanoparticles had no therapeutic effect when administered alone (**a**) EAE mice were intravenously injected with MSN-Ce (4 mg cerium/kg), CeNPs (4 mg cerium/kg), or left untreated on days 30, 34, and 38 after EAE induction (n = 3 biologically independent animals), (**b**) clinical score was recorded until day 45. (**c**) EAE mice were intravenously injected with MSN (90 μg) and MSN-Ce (4 mg cerium/kg) on days 12 and 15 after EAE induction (n = 5 biologically independent animals), (**d**) EAE clinical score. (**e**) EAE mice were intravenously injected with pegylated CeNPs (4 mg cerium/kg) on days 4, 7, and 10 after EAE induction or left untreated. n = 5 (untreated) or 6 (pegylated CeNPs) biologically independent animals, (**f**) EAE clinical score. The data in (**b**) are represented as mean ± SD and were subjected to a one-way ANOVA with Dunnett's multiple comparisons test. The data in (**d**, **f**) are represented as mean ± SE. The data in (**d**, **f**) were analyzed by an unpaired two-tailed t-test. $P < 0.05$ was considered significant, ns = not significant.

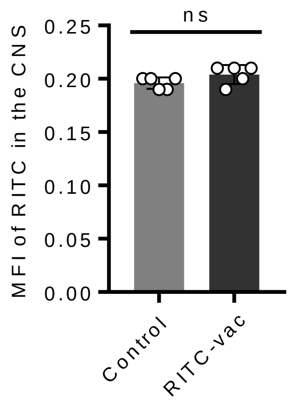

Supplementary Figure 16. Nanovaccine distribution in the CNS. Mean fluorescence intensity of RITC in cells isolated from spinal cord of EAE mice that were intravenously injected with MSN-MOG-Ce loading RITC (RITC-Vac) or left untreated (control), n = 5 biologically independent animals. The data are represented as mean ± SD and were subjected to an unpaired two-tailed t-test, ns = not significant.

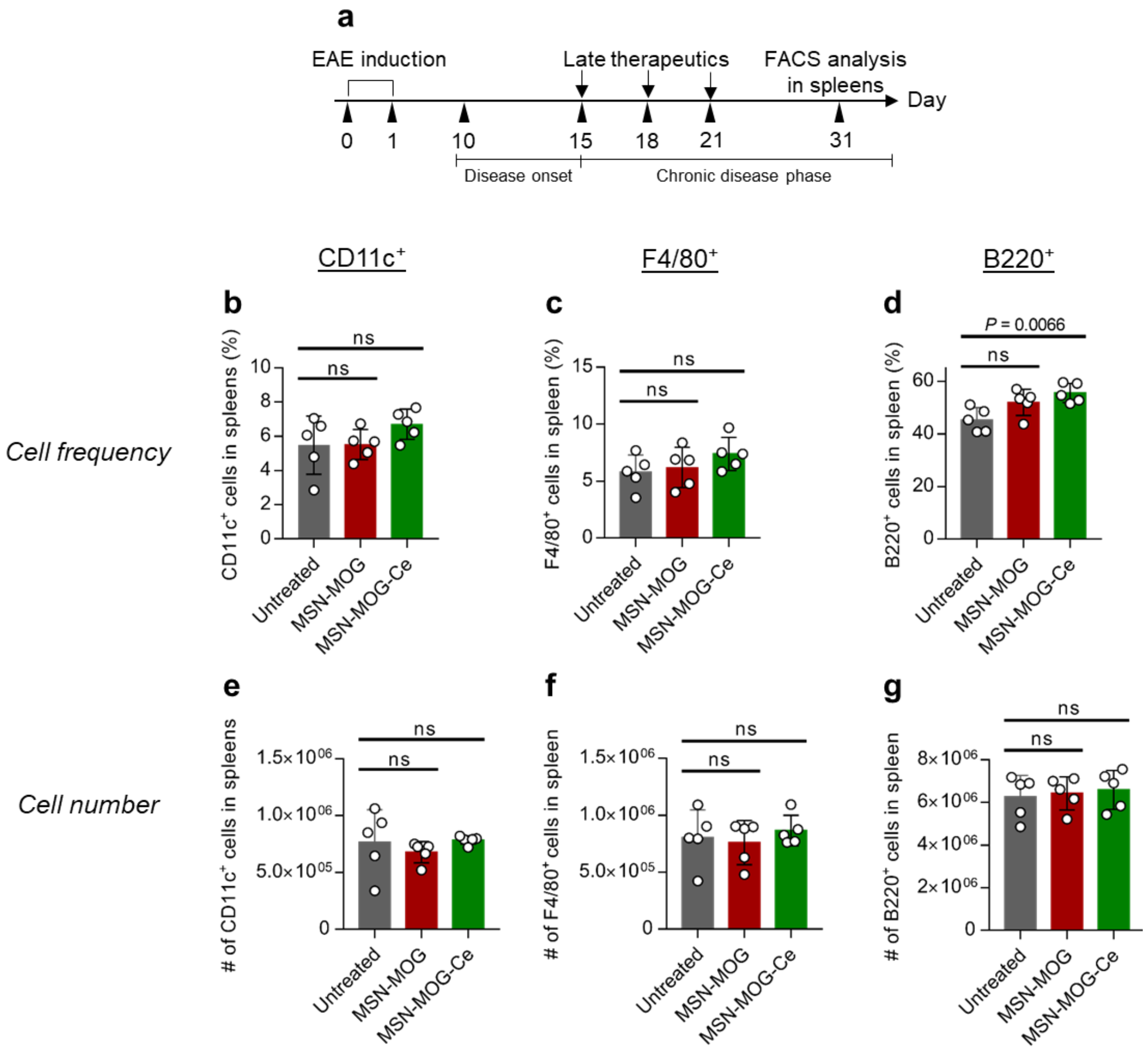

Supplementary Figure 17. Analysis of APCs in spleens after administering late therapeutics (**a**) EAE was induced in C57BL/6 mice, followed by thrice intravenous injection of MSN-MOG or MSN-MOG-Ce, or left untreated, on days 15, 18, and 21 prior to flow cytometry analysis of splenocytes on day 31; n = 5 biologically independent animals. (**b**, **c, d**) The frequencies of CD11c$^+$, F4/80$^+$, and B220$^+$ cells, respectively. (, **e, f, g**) The numbers of CD11c$^+$, F4/80$^+$, and B220$^+$ cells, respectively. The data in (**b-g**) are represented as mean ± SD and were subjected to a one-way ANOVA with Dunnett's multiple comparisons tests. P < 0.05 was considered significant, ns = not significant

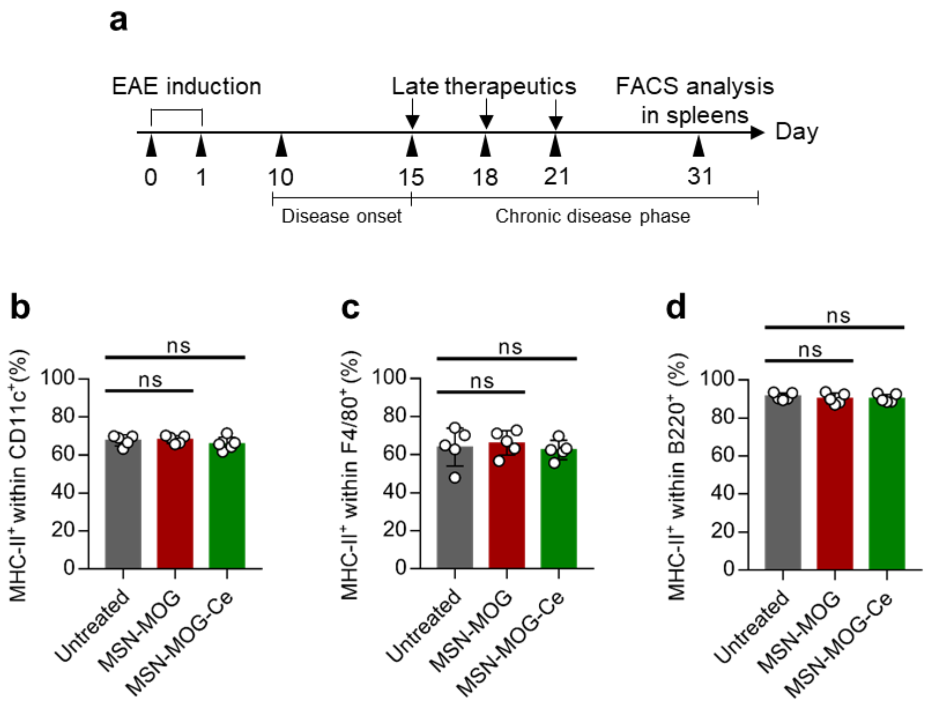

Supplementary Figure 18. Analysis of MHC-II expression on APCs in spleens after administering late therapeutics (**a**) EAE was induced in C57BL/6 mice, followed by intravenous injection of MSN-MOG, MSN-MOG-Ce, or left untreated, on days 15, 18, and 21 prior to flow cytometry analysis of splenocytes on day 31; n = 5 biologically independent animals. (**b**, **c**, **d**) The expression of MHC-II on CD11c$^+$ DCs, F4/80$^+$ macrophages, and B220$^+$ cells, respectively. Data in (**b-d**) are represented as mean ± SD and were subjected to a one-way ANOVA with Dunnett's multiple comparisons tests, ns = not significant.

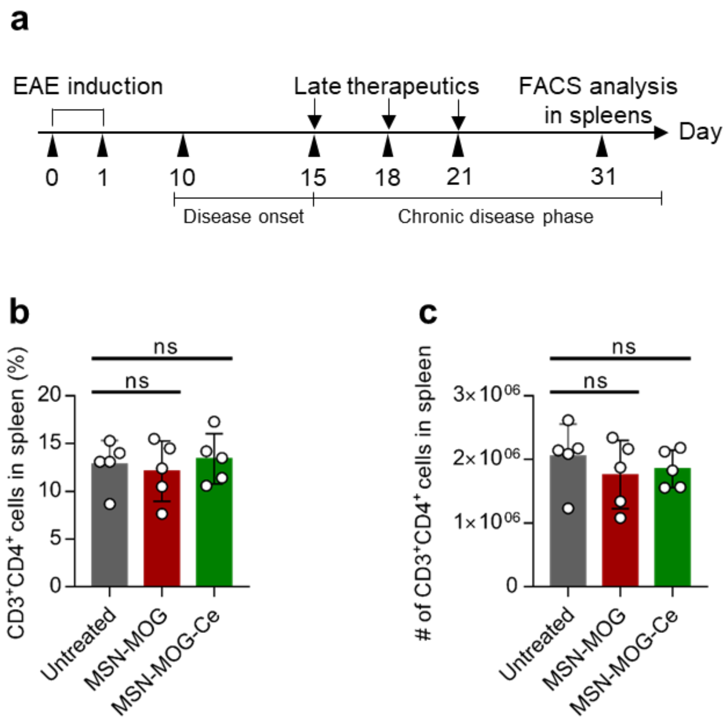

Supplementary Figure 19. Analysis of T-cells in the spleens after treatment with late therapeutics (a) EAE was induced in C57BL/6 mice, followed by intravenous injection of MSN-MOG, MSN-MOG-Ce, or left untreated, on days 15, 18, and 21 prior to flow cytometry analysis of splenocytes on day 31; n = 5 biologically independent animals. (**b**, **c**) The frequency and number of $CD4^+$ T-cells, respectively, in the spleens. Data in (**b** and **c**) are represented as mean ± SD and were subjected to a one-way ANOVA with Dunnett's multiple comparisons tests, ns = not significant.

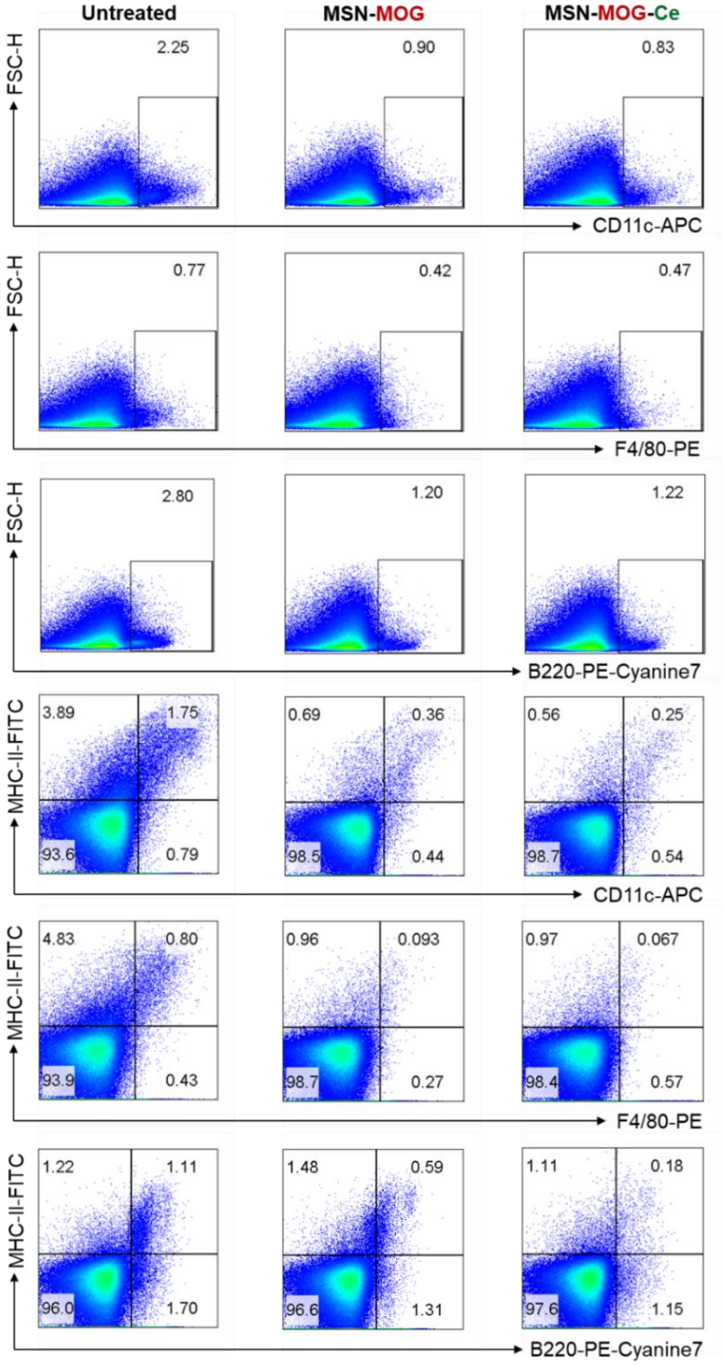

Supplementary Figure 20. MSN-MOG-Ce vaccine suppressed infiltration of APCs into CNS and their antigen presentation capacity in late therapeutics. Representative pseudocolor plots showing the percentages of $CD11c^+$ cells, $F4/80^+$ cells, $B220^+$ cells, and MHC-II expression on the cells in the CNS of EAE mice on day 31 after late therapeutics study with MSN-MOG and MSN-MOG-Ce. The numbers in the plots depict the percentage.

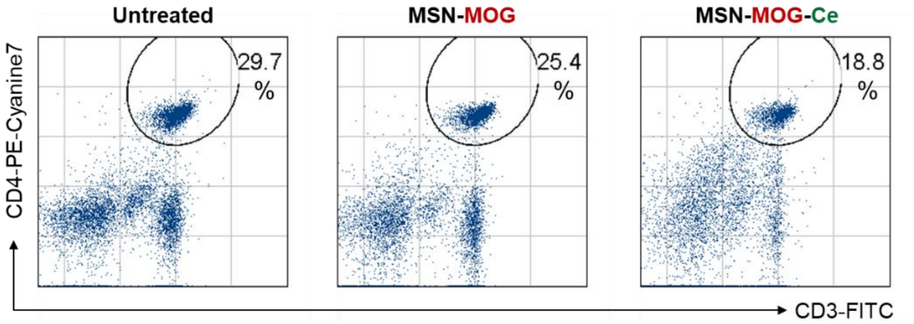

Supplementary Figure 21. MSN-MOG-Ce vaccine inhibited CD4$^+$ T-cell in the CNS-draining lymph node in late therapeutics. Representative plots of frequency of CD4$^+$ T-cell in the cervical lymph node in EAE mice after late therapeutics with MSN-MOG and MSN-MOG-Ce.

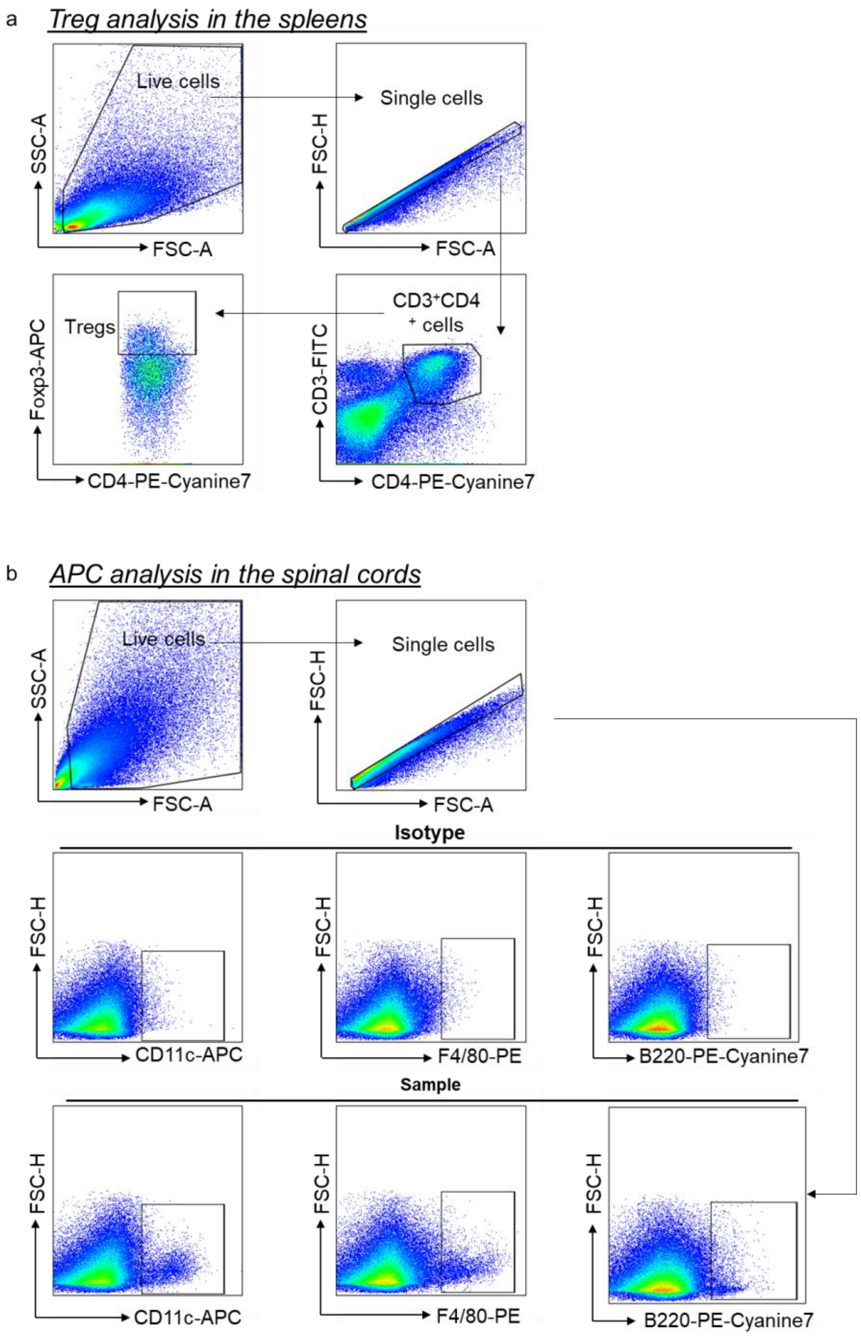

Supplementary Figure 22. Gating strategies for flow cytometry (**a**) Gating strategy to sort Treg ($CD3^+CD4^+Foxp3^+$) cells from splenocytes of EAE mice (C57BL/6) that were immunized with different groups of vaccine. (**b**) Gating strategy to sort APCs ($CD11c^+$, $F4/80^+$, and $B220^+$ cells) from spinal cords of EAE mice (C57BL/6) that were immunized with different groups of vaccine.

Supplementary Movie 1. Movement recovery of EAE-induced animals after late-therapeutic treatment of MSN-MOG EAE was induced in C57BL/6 mice on days 0 and 1, which was followed by intravenous injection of MSN-MOG on days 15, 18, and 21 (n = 5). The video showing the movement of a representative animal on day 15 after EAE induction (before the first injection, upper) and its movement on day 22 (after 3 injections, lower).

Supplementary Methods

Zeta potential measurement: Zeta potential of the nanoparticle was measured using a Zetasizer Nano ZS90 (Malvern Panalytical, UK) at 25°C in DI water and PBS.

***Degradation test*:** PBS was used to disperse MSN (1 mg/mL) and MSN-MOG-Ce (1 mg/mL) at 37 °C before being analyzed by TEM (JEM-2100F, JEOL, Akishima, Japan) at indicated time points.

***Fourier transform infrared spectroscopy (FTIR) measurement*:** Dried form of the nanoparticles was measured by FTIR spectrophotometer (Secondary Nicolet iZ10, Thermo Fisher Scientific, USA) which is connected to an attenuated total reflectance accessory with zinc-selenide crystal in the wavenumber range 4000-400 cm^{-1} at the resolution of 4 cm^{-1} and the scan number of 32.

In vitro release profile: MOG peptide was loaded in MSN with or without the adsorption of CeNPs. The resulted MSN-MOG and MSN-MOG-Ce were resuspended in PBS under gentle shaking. The supernatant was collected and refreshed periodically. The released MOG and cerium were measured by micro BCA protein assay kit (Thermo Scientific) and ICP-OES, respectively.

Nanoparticle distribution in CNS: RITC-MSNs were used to load MOG and CeNPs before being administered intravenously into EAE mice three times with 3 days interval (90 µg MSN/mouse/time). 3 days after the last injection, cells from the spinal cords were isolated for the detection of RITC signal by flow cytometry.
