# Supplementary material for: Immunosuppressive biomaterial-based therapeutic vaccine to treat multiple sclerosis via re-establishing immune tolerance
Source: Nat Commun. 2022 Dec 2;13:7449. doi: 10.1038/s41467-022-35263-9 (PMC9718828; doi:10.1038/s41467-022-35263-9)
Supplement: Supplementary file 2 — Description of Additional Supplementary Files [file 41467_2022_35263_MOESM2_ESM.pdf]

Description of Additional Supplementary Files

File Name: Supplementary Movie 1
Description: **Movement recovery of EAE-induced animals after late-therapeutic treatment of MSN-MOG** EAE was induced in C57BL/6 mice on days 0 and 1, which was followed by intravenous injection of MSN-MOG on days 15, 18, and 21 (n = 5). The video showing the movement of a representative animal on day 15 after EAE induction (before the first injection, upper) and its movement on day 22 (after 3 injections, lower).
