# Supplementary material for: Immunosuppressive biomaterial-based therapeutic vaccine to treat multiple sclerosis via re-establishing immune tolerance
Source: Nat Commun. 2022 Dec 2;13:7449. doi: 10.1038/s41467-022-35263-9 (PMC9718828; doi:10.1038/s41467-022-35263-9)
Supplement: Supplementary file 4 — Reporting Summary [file 41467_2022_35263_MOESM4_ESM.pdf]

nature portfolio

Corresponding author(s): Jaeyun Kim

Last updated by author(s): Nov 20, 2022

Reporting Summary

Nature Portfolio wishes to improve the reproducibility of the work that we publish. This form provides structure for consistency and transparency in reporting. For further information on Nature Portfolio policies, see our Editorial Policies and the Editorial Policy Checklist.

Statistics

For all statistical analyses, confirm that the following items are present in the figure legend, table legend, main text, or Methods section.

n/a	Confirmed	
☐	☒	The exact sample size (*n*) for each experimental group/condition, given as a discrete number and unit of measurement
☐	☒	A statement on whether measurements were taken from distinct samples or whether the same sample was measured repeatedly
☐	☒	The statistical test(s) used AND whether they are one- or two-sided<br>*Only common tests should be described solely by name; describe more complex techniques in the Methods section.*
☒	☐	A description of all covariates tested
☒	☐	A description of any assumptions or corrections, such as tests of normality and adjustment for multiple comparisons
☐	☒	A full description of the statistical parameters including central tendency (e.g. means) or other basic estimates (e.g. regression coefficient) AND variation (e.g. standard deviation) or associated estimates of uncertainty (e.g. confidence intervals)
☐	☒	For null hypothesis testing, the test statistic (e.g. *F*, *t*, *r*) with confidence intervals, effect sizes, degrees of freedom and *P* value noted<br>*Give P values as exact values whenever suitable.*
☒	☐	For Bayesian analysis, information on the choice of priors and Markov chain Monte Carlo settings
☒	☐	For hierarchical and complex designs, identification of the appropriate level for tests and full reporting of outcomes
☒	☐	Estimates of effect sizes (e.g. Cohen's *d*, Pearson's *r*), indicating how they were calculated

Our web collection on statistics for biologists contains articles on many of the points above.

Software and code

Policy information about availability of computer code

Data collection	Flow-cytometry data were acquired via MACSQuant VYB flow cytometer (Miltenyi Biotec, version No. 2.6.1515.13751) and were analyzed in FlowJo v10.0 software. Optical microscopy data were collected via (ECLIPSE Ti–U, Nikon, Japan. No custom code was used to collect data
Data analysis	Statistical analysis was performed by using GraphPad Prism v7. GraphPad Prism 7.00, Microsoft Excel 2013, and Microsoft PowerPoint 2013 for Windows were used to draw graphs and schemes

For manuscripts utilizing custom algorithms or software that are central to the research but not yet described in published literature, software must be made available to editors and reviewers. We strongly encourage code deposition in a community repository (e.g. GitHub). See the Nature Portfolio guidelines for submitting code & software for further information.

Data

Policy information about availability of data

All manuscripts must include a data availability statement. This statement should provide the following information, where applicable:

- Accession codes, unique identifiers, or web links for publicly available datasets
- A description of any restrictions on data availability
- For clinical datasets or third party data, please ensure that the statement adheres to our policy

The authors declare that all data supporting the results are provided within the manuscript in the Source Data file.

Field-specific reporting

Please select the one below that is the best fit for your research. If you are not sure, read the appropriate sections before making your selection.

☒ Life sciences ☐ Behavioural & social sciences ☐ Ecological, evolutionary & environmental sciences

For a reference copy of the document with all sections, see nature.com/documents/nr-reporting-summary-flat.pdf

Life sciences study design

All studies must disclose on these points even when the disclosure is negative.

Sample size	The sample size was determined based on previous work [1], and was given based on consultation with veterinary staff and SungKyunKwan University IACUC to ensure sufficient power for statistical analysis while minimizing the number of animals used. No statistical method was used to predetermine the sample size.<br><br>This paper [1] "Design of biodegradable nanoparticles to modulate phenotypes of antigen-presenting cells for antigen-specific treatment of autoimmune disease." Biomaterials 222 (2019): 119432 was used as reference to determine sample size
Data exclusions	No data were excluded for in vitro and in vivo studies
Replication	The experimental results were reliably reproduced. Late therapeutic treatment of EAE were performed independently at least twice by independent investigators. Representative experiments, representative micrographs, and in vitro tests were repeated independently at least twice with biologically independent samples.
Randomization	All samples/organisms were randomly allocated into experimental groups. Randomization was conducted in semi-therapeutic treatment of EAE mice. Mice from the same litter were induced with EAE and randomly distributed in cages prior to vaccination. For early and late therapeutic studies, only successful EAE-induced mice were used at the time of vaccination
Blinding	For all EAE treatment studies, EAE clinical scores were recorded by two independent investigators in blinded manner and the investigators were blinded to group allocation during data collection

Reporting for specific materials, systems and methods

We require information from authors about some types of materials, experimental systems and methods used in many studies. Here, indicate whether each material, system or method listed is relevant to your study. If you are not sure if a list item applies to your research, read the appropriate section before selecting a response.

Materials & experimental systems

n/a	Involved in the study
☐	☒ Antibodies
☐	☒ Eukaryotic cell lines
☒	☐ Palaeontology and archaeology
☐	☒ Animals and other organisms
☒	☐ Human research participants
☒	☐ Clinical data
☒	☐ Dual use research of concern

Methods

n/a	Involved in the study
☒	☐ ChIP-seq
☐	☒ Flow cytometry
☒	☐ MRI-based neuroimaging

Antibodies

Antibodies used

Antibody, Supplier, Catalogue number, Clone; Isotype

FcR Blocking Reagent mouse, Miltenyi Biotec, 130-092-575
Anti-mouse IFN-gamma, Miltenyi Biotec, 130-117-780, REA638
Anti-mouse IL-10, Miltenyi Biotec, 130-102-349, JES5-16E3; Rat IgG2b kappa Isotype Control
Anti-mouse IL-17A, Miltenyi Biotec, 130-103-015, TC11-18H10; Rat IgG1 kappa Isotype Control
Anti-mouse CD4, Miltenyi Biotec, 130-094-164, GK1.5; Rat IgG2b Isotype Control (Treg Detection Kit)
Anti-mouse CD25, Miltenyi Biotec, 130-094-164, 7D4; Rat IgM Isotype Control (Treg Detection Kit)
Anti-mouse Foxp3, Miltenyi Biotec, 130-094-164, 3G3; Rat Ig1 Isotype Control (Treg Detection Kit)
Anti-mouse CD11c, eBioscience, 17-0114-82, N418; Armenian Hamster IgG Control, eBioscience, 17-4888-81
Anti-mouse F4/80, eBioscience, 48-4801-80, BM8; Rat IgG2a kappa Isotype Control, eBioscience, 48-4801-80
Anti-mouse F4/80, eBioscience, 12-4801-82, BM8; Rat IgG2a kappa Isotype Control, eBioscience, 12-4321-80
Anti-mouse B220 (CD45R), eBioscience, 25-0452-81, RA3-6B2; Rat IgG2a kappa Isotype Control, eBioscience, 25-4321-81
Anti-mouse MHC-II, eBioscience, 11-5321-82, M5/114.15.2; Rat IgG2b kappa Isotype Control, eBioscience, 11-4031-81
Anti-mouse CD86, eBioscience, 48-0862-82, GL1; Rat IgG2a kappa Isotype Control, eBioscience, 48-4321-80

Anti-mouse CD40, eBioscience, 11-0402-81, HM40-3; Armenian hamster IgM kappa Isotype Control
Anti-mouse CD3, eBioscience, 11-0032-82, 17A2; Rat IgG2b kappa Isotype Control, eBioscience, 11-4031-81
Anti-mouse CD4, eBioscience, 25-0041-82, GK1.5; Rat IgG2b kappa Isotype Control, eBioscience, 25-4031-81
Anti-mouse Foxp3, eBioscience, 17-5773-82, FJK-16s; Rat IgG2a kappa Isotype Control, eBioscience, 17-4321-81
Anti-mouse TCR Vα2, BioLegend, 127815, B20.1; Rat IgG2a lamda Isotype Control
Anti-Iba1/AIF-1, Cell Signaling Technology, 78060S, E4O4W
I-Ab MOG 35-55 Tetramer, MBL, TS-M704-1

Validation

All antibodies were purchased from the supplier as noted above and the dilution factor was determined following the manufacturer's instruction. The antibodies were all used for flow cytometric analysis of murine cells.

FcR Blocking Reagent mouse: https://www.miltenyibiotec.com/upload/assets/IM0001509.PDF

Anti-mouse IFN-gamma: https://www.miltenyibiotec.com/upload/assets/dataSheet_p67611_eng_GBR.pdf

Anti-mouse IL-10: https://www.miltenyibiotec.com/_Resources/Persistent/0d9244d4808302e18863e06a4157a7a7388e1590/DS_IL-10_Antibody_anti-mouse_APC_JES5-16E3_130-102-349.pdf

Anti-mouse IL-17A: https://www.miltenyibiotec.com/upload/assets/dataSheet_p26778_eng_GBR.pdf

Anti-mouse CD4, Anti-mouse CD25, Anti-mouse Foxp3: https://www.miltenyibiotec.com/_Resources/Persistent/944a5f6d7bdee92335a4dc9895f3a16998b2ea98/DS_130-094-164.pdf

Anti-mouse CD11c: https://www.thermofisher.com/antibody/product/CD11c-Antibody-clone-N418-Monoclonal/17-0114-82

Anti-mouse F4/80: https://www.thermofisher.com/antibody/product/F4-80-Antibody-clone-BM8-Monoclonal/48-4801-82, https://www.thermofisher.com/antibody/product/F4-80-Antibody-clone-BM8-Monoclonal/12-4801-82

Anti-mouse B220 (CD45R): https://www.thermofisher.com/antibody/product/CD45R-B220-Antibody-clone-RA3-6B2-Monoclonal/25-0452-82

Anti-mouse MHC-II: https://www.thermofisher.com/antibody/product/MHC-Class-II-I-A-I-E-Antibody-clone-M5-114-15-2-Monoclonal/11-5321-82

Anti-mouse CD86: https://www.thermofisher.com/antibody/product/CD86-B7-2-Antibody-clone-GL1-Monoclonal/48-0862-82

Anti-mouse CD40: https://www.thermofisher.com/antibody/product/CD40-Antibody-clone-HM40-3-Monoclonal/11-0402-82

Anti-mouse CD3: https://www.thermofisher.com/antibody/product/CD3-Antibody-clone-17A2-Monoclonal/11-0032-82

Anti-mouse CD4: https://www.thermofisher.com/antibody/product/CD4-Antibody-clone-GK1-5-Monoclonal/25-0041-82

Anti-mouse Foxp3: https://www.thermofisher.com/antibody/product/FOXP3-Antibody-clone-FJK-16s-Monoclonal/17-5773-82

Anti-mouse TCR Vα2: https://production-dynamicweb.biolegend.com/fr-ch/products/pacific-blue-anti-mouse-tcr-valpha2-antibody-6657

Anti-Iba1/AIF-1: https://www.cellsignal.com/datasheet.jsp?productId=78060&images=1&size=A4

I-Ab MOG 35-55 Tetramer: https://www.mblintl.com/products/wp-content/uploads/sites/2/2020/08/TS-M704-1-v3.pdf

Eukaryotic cell lines

Policy information about cell lines

Cell line source(s)

RAW264.7 - ATCC

Authentication

By checking cell morphology on microscope and checking cell surface markers via FACS.

Mycoplasma contamination

Mycoplasma contamination was not detected in the cells before using

Commonly misidentified lines
(See ICLAC register)

No commonly misidentified cell lines were used

Animals and other organisms

Policy information about studies involving animals; ARRIVE guidelines recommended for reporting animal research

Laboratory animals

Female C57BL/6 mice (OrientBio, Seongnam, South Korea) between 10 weeks and 11 weeks old were used for all EAE experiments.
7-9 week age, female OT-II (C57BL/6-Tg(TcraTcrb)425Cbn/Crl) mice were used for in vitro generation of Treg cells
Dark/light cycle : light 8am-8pm. Ambient temperature : 23 +/- 2 degree Celcius. Humidity : 40~60%
The experimental/control animals were co-housed under the specific pathogen-free condition and euthanized by carbon dioxide (flow rate: 3 L/min) at the end of the studies

Wild animals	No wild animal was used in this study
Field-collected samples	The study did not involve samples collected from the field
Ethics oversight	SungKyunKwan University's Institutional Animal Care and Use Committee (SKKUIACUC, No. 2020-01-15-1)

Note that full information on the approval of the study protocol must also be provided in the manuscript.

Flow Cytometry

Plots

Confirm that:

☒ The axis labels state the marker and fluorochrome used (e.g. CD4-FITC).

☒ The axis scales are clearly visible. Include numbers along axes only for bottom left plot of group (a 'group' is an analysis of identical markers).

☒ All plots are contour plots with outliers or pseudocolor plots.

☒ A numerical value for number of cells or percentage (with statistics) is provided.

Methodology

Sample preparation	For spleen samples, they were processed through mechanical disruption and filtered through 70-µm cell strainer. Cells were then centrifuged for 5 min, 2000 rpm, 4 °C lysed by ammonium-chloride-potassium (ACK) lysing buffer (Lonza), then the collected cells were washed twice by cold PBS<br><br>Spinal cords were dissociated in cold PBS containing 1 mg/mL collagenase type IV and 20% EDTA/trypsin and incubated at 37 °C for 20 min. RPMI 1640 containing 10% fetal bovine serum was added to each sample to inhibit enzymatic activity, and the cells were filtered through a 40-µm cell strainer. The cells were collected by centrifugation according standard protocol<br><br>The isolated cells were first stained with FcR blocking reagent, followed by antibodies against surface antigens. For transcription factor staining, cells were subsequently fixed, permeabilized by Foxp3/Transcription Factor staining buffer set (eBioscience) and stained with antibody against Foxp3. All antibodies were diluted according to manufacturer's recommendations
Instrument	Stained cells were analyzed by a MACSQuant VYB flow cytometer (Miltenyi Biotec)
Software	Treestar FlowJo v10.0 was used to gate the samples and Graphpad was used for statistical analysis
Cell population abundance	At least 60,000 total events were acquired for in vitro FACS analysis of BMDC activation and ROS scavenging experiments. At least 300,000 total events were acquired for FACS analysis in vivo
Gating strategy	All cells were gated based on forward-scatter and side-scatter characteristics to exclude dead cells and debris. Singlet cells were gated using FSC-H and FSC-A. Subsequently, cells were gated based on isotype controls, and the frequencies of positively stained cells for each marker was recorded

☒ Tick this box to confirm that a figure exemplifying the gating strategy is provided in the Supplementary Information.
